# Analysis of several anionic polar pesticides in food of plant and animal origin using QuPPe extraction and CE-MS/MS determination

**DOI:** 10.1007/s00216-025-05990-1

**Published:** 2025-07-21

**Authors:** Ann-Kathrin Schäfer, Walter Vetter, Michelangelo Anastassiades

**Affiliations:** 1 Section for Residues and Contaminants, Chemisches Und Veterinäruntersuchungsamt Stuttgart, 70736 Fellbach, Germany; 2 Institute of Food Chemistry (170b), University of Hohenheim, 70599 Stuttgart, Germany

**Keywords:** Capillary electrophoresis, Mass spectrometry, CE-MS/MS, Anionic polar pesticide, QuPPe, Glyphosate

## Abstract

Although highly polar pesticides such as glyphosate (Gly) are widely used and highly relevant for pesticide residue analysis, their analytical determination is still problematic. Methods involving liquid chromatography in combination with tandem mass spectrometry (LC-MS/MS) often lack sensitivity due to insufficient separation from matrix co-extractives causing severe ion suppression effects on Gly and other anionic pesticides. In this study, capillary electrophoresis (CE) was used for an improved separation of analytes from matrix components, thus increasing sensitivity and robustness of analysis. Gly and 13 other anionic pesticides and selected metabolites (including glufosinate, ethephon, fosetyl, phosphonic acid and chlorate) were studied after extraction with the quick polar pesticides (QuPPe) method. Different tests resulted in an improved separation and sensitivity using a background electrolyte (BGE) containing 15% acetic acid and 20% methanol (MeOH) in water. The study also included the investigation of injection conditions and a thorough study of the matrix effects. Finally, results of method validation in several (strawberry, soybean, milk, liver, lemon) matrices of plant and animal origin are presented.

**Graphical Abstract:**

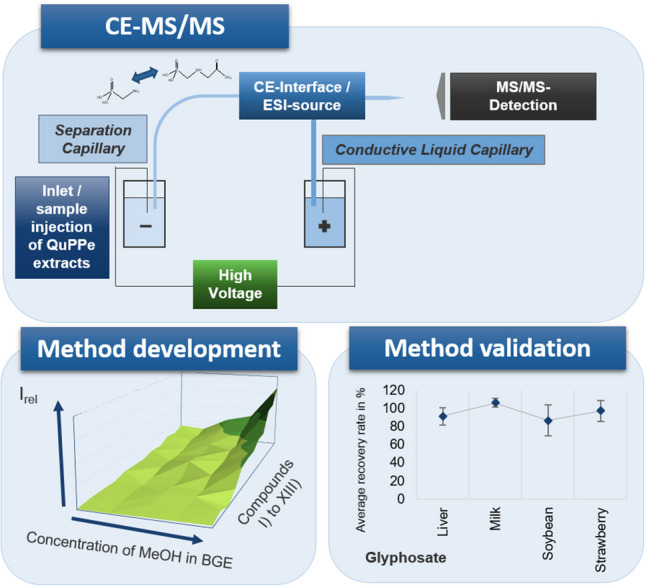

**Supplementary Information:**

The online version contains supplementary material available at 10.1007/s00216-025-05990-1.

## Introduction

Glyphosate (Gly) is currently the most heavily applied herbicide [1, 2]. Gly is used in weed control of annual or perennial species, and as a desiccant to facilitate the harvest of essential food crops [1]. In 2022, about 2% of the officially controlled samples in the European Union (EU) contained Gly at quantifiable levels and 0.3% of the samples even exceeded the maximum residue level (MRL) [3]. Although, its use for desiccation is not authorised within the EU since the renewal of the active substance in 2023, its approval in the EU as a broad-band herbicide has been extended until 2033 [4]. Therefore, Gly is still a major target analyte of food control institutions when monitoring compliance of residue levels with current regulations. However, the analysis of Gly and its important metabolite aminomethylphosphonic acid (AMPA) is challenging due to their high polarity and the presence of ionisable moieties (list of abbreviations, Supplementary Table S1). The traditional analysis of Gly and AMPA involved derivatization, e.g. by fluorenylmethyloxycarbonyl chloride (Fmoc-Cl), followed by high-performance liquid chromatography (HPLC) or LC, or gas chromatography (GC) combined with various detection approaches including tandem mass spectrometry (MS/MS; LC- or GC-MS/MS) [5–7].

Besides being cumbersome and sometimes error-prone, methods involving derivatization do not cover phosphorous-containing analytes lacking a secondary amine group such as *N*-acetyl metabolites of Gly and glufosinate [8]. Therefore, LC-MS/MS [8–10] and ion chromatography (IC) with high-resolution mass spectrometry (IC-HRMS) or MS/MS (IC-MS/MS) [11–13] analysis methods of free Gly, AMPA and other polar compounds have gained popularity. However, these direct chromatographic methods often suffer from a lack of sensitivity and the impact of matrix on ion suppression in the ion source and/or retention time shifts [8, 10].

As capillary electrophoresis (CE) - usually performed as capillary zone electrophoresis (CZE) - involves a completely different separation mechanism than LC or IC, it appears to be a promising alternative for avoiding the matrix effect–related limitations of the other two techniques [14]. In brief, CE separations are based on individual electrophoretic mobilities of charged molecules [15, 16]. In CE, a high voltage is applied to generate an electric field that induces the movement of analytes [15–17], embedded in a background electrolyte (BGE) via a non-equilibrium separation mechanism [17, 18]. CE uses narrow capillaries filled with BGE, and involves small liquid flows, and thus does not require intensive liquid evaporation when coupling with mass spectrometry (MS) and MS/MS as in LC-MS/MS [15]. Various interfaces with (sheath) and without admixture of liquids (sheathless) can be used to connect CE with MS, through an electrospray ionization (ESI) interface [15, 17–19]. CE has been successfully applied for the analysis of neonicotinoids [20], phenoxy acid herbicides [21], triazines [22], as well as Gly and other analytes with various detection systems [23–29] including MS, MS/MS [30–32] or time-of-flight mass spectrometry (TOF-MS) [14, 33]. The first studies using CE already indicated an efficient separation of matrix from Gly [14, 23].

The present study aimed at developing and validating a CE-MS/MS method for the direct determination of residues of Gly and 13 further anionic polar pesticides, metabolites, and contaminants in food of plant and animal origin. Samples were extracted with the Quick Polar Pesticides (QuPPe) method for highly polar pesticides initially developed by the European Reference Laboratory for Pesticides requiring Single Residue Methods (EURL-SRM) [9]. In this procedure, isotope-labelled internal standards (IL-ISs) were employed to correct matrix effects and other sources of errors and therefore improve the method’s precision and accuracy [9, 34]. This study included the investigation of the BGE composition and the injection conditions for the separation of the analytes, as well as matrix effects and their influence on the analysis.

## Experimental

### Chemicals and samples

Methanol (MeOH) and acetonitrile (ACN) for sample preparation (MS grade) were purchased from Th. Geyer (Renningen, Germany), Merck/Supelco (Darmstadt, Germany) or Biosolve Chemicals (Valkenswaard, The Netherlands) (different sources due to supply difficulties). 2-Propanol (MS grade) was obtained from Merck/Supelco (Darmstadt, Germany). Formic acid (FA) for sample preparation was obtained from Merck (Darmstadt, Germany) or Honeywell (Charlotte, NC, USA). Formic acid and acetic acid (HAc) in MS grade were purchased from Merck/LiChropur (Darmstadt, Germany) or LGC/Fluka (Teddington, England). Ammonium formate (LC-MS grade) was ordered from LGC/Fluka (Teddington, England) and ammonium acetate (NH_4_Ac) at LC-MS grade from Merck/Sigma-Aldrich (Darmstadt, Germany). Ethylenediaminetetraacetic acid tetrasodium (Na_4_EDTA × 4H_2_O) for extraction was obtained from Merck/Millipore (Darmstadt, Germany), and C_18_ sorbent (POLYGOPREP 300–30 C18, particle size 30 µm, pore size 300 Å) for sample preparation originated from Macherey–Nagel (Düren, Germany). Chemicals for IL-IS synthesis were potassium chloride (Merck/Millipore, Darmstadt, Germany), phosphorous trichloride (Merck/Sigma-Aldrich, Darmstadt, Germany) and H_2_^18^O (Medical Isotopes, Pelham, NH, USA).

Standard substances of native analytes and the corresponding IL-ISs were delivered from HPC (Cunnersdorf, Germany), LGC (Toronto Research Chemical (TRC) or Fluka or Dr. Ehrenstorfer, Teddington, England), Merck/Sigma-Aldrich (Darmstadt, Germany), Merck (Darmstadt, Germany) and ASCA (Adlershof, Germany). IL-IS of chlorate and perchlorate were synthesized electrochemically by oxidation of chloride in H_2_^18^O in our laboratory (Supplementary Table S2). Similarly, the IL-IS of phosphonic acid was synthesized by hydrolysis of phosphorous trichloride in H_2_^18^O in our laboratory. Stock solutions were prepared at 1000 µg/ml in water/ACN (90/10 v/v), except for fosetyl and its IL-IS (100 µg/ml in water/ACN (90/10 v/v), due to solubility issues). Stock solutions of ethephon and its IL-IS additionally contained 0.1% hydrochloric acid (HCl). Samples of strawberry, lemon, cucumber, rice, soybean, milk, liver and kidney were obtained from the local market while the Swiss chard was grown in a private garden.

### Instrumentation

The CE system consisted of a Sciex CESI 8000 Plus ESI device (AB Sciex, Framingham, MA, USA) equipped with a NanoSpray III Source (AB Sciex, Framingham, MA, USA) interface and an AB Sciex QTrap 5500 mass spectrometer (AB Sciex, Framingham, MA, USA). The CE system was monitored with CESI 8000 Plus Software (Beckman Coulter, Brea, CA, USA) and the MS with Analyst software (AB Sciex, Framingham, MA, USA). A Beckman Coulter OptiMS silica surface cartridge was used as the separation capillary (Beckman Coulter, Brea, CA, USA). The separation was run with 30 kV constant voltage in reversed polarity for 16 min [35]. A composite of HAc/MeOH/water (15/20/65, v/v/v, pH 2.2) was used as BGE and HAc/water (10/90, v/v) as conductive liquid [35]. The latter is used in order to establish a closed circuit with the employed interface and does not come into contact with the analytes. Sample extracts were diluted fivefold with MeOH_FA_/H_2_O (7/3, v/v; with MeOH_FA_ being MeOH containing 1% formic acid), and as focusing buffer (for sample stacking during injection), an aqueous 5 mM NH_4_Ac solution (pH 6.7) was used. Glufosinate was the exception requiring the use of the BGE (see 'Validation of the CE-MS/MS method') for dilution while the focusing buffer was omitted for glufosinate and AMPA [35]. Before each run, the system was rinsed with (i) 0.1 M NaOH, (ii) 0.1 M HCl, (iii) BGE and (iv) conductive liquid (2 min each) [35]; see also Supplementary Table S3 for more details on instrumentation. In CE-MS/MS, two or three mass transitions and one transition for the corresponding IL-ISs were recorded for each analyte (Supplementary Tables S2 and S4). In order to gain more sensitivity, the dwell time was set at reasonably high levels (typically between 30 and 50 ms), refraining from acquiring a second mass transition for the IL-IS. Fortunately, the CE electropherograms of the acquired IL-IS transitions did not show any notable matrix signals at the migration times of the respective analytes.

### Sample preparation with the QuPPe method

Samples of plant origin (PO) were prepared as described in the QuPPe-PO method and those of animal origin (AO) as described in the QuPPe-AO method [9, 36]. In brief, typically 5 g or 10 g of the homogenized samples were extracted with MeOH_FA_ (MeOH containing 1% formic acid) [9]. After a freeze-out step and centrifugation, the extracts were filtered before the measurement [9]. For AO matrices and PO matrices with a relatively high protein content, like cereals, pulses and oily seeds (here, rice and soybean), the extraction involved protein precipitation by adding additional formic acid during extraction and by additionally diluting the isolated extract with ACN (1:1, v/v) [9, 36]. Furthermore, EDTA was added during extraction for the complexation of metal cations, to reduce their interaction with analytes that tend to form metal chelates, such as Gly and AMPA [9, 36]. The above-mentioned ACN precipitation was combined with a dispersive solid-phase extraction (dSPE) step using octadecylsilane (ODS) sorbent [9, 36].

Matrix effects were studied using residue-free matrix extracts prepared without adding IL-ISs. These extracts contained no detectable levels of any of the analytes of interest except trace levels of phosphonate. The bovine liver contained high natural levels of 2-hydroxyethylphosphonic acid (HEPA) [37] so this compound was exempted from validation in this matrix. Further modifications are described in each experiment.

### Investigations on the composition of the BGE

To elaborate the separation conditions in CE, the composition of the BGE was initially varied, while keeping the composition of the conductive liquid constant at 10% HAc in water. HAc as a component in BGE was tested at 1%, 5%, 10% and 15% in water. BGE compositions entailing 15% HAc in water were then further examined in combination with organic solvents, to gain higher ionization yields and signal intensities by facilitating evaporation within the ion source. Combinations of 15% HAc with MeOH (5%, 10%, 20% or 50%) or ACN (20%) were tested alongside HAc (5% and 10%) with 50 mM NH_4_Ac.

Standard solutions were prepared in (i) pure BGE/solvent (at the respective composition of each experiment) and (ii) QuPPe [9] blank matrix extracts of Swiss chard after dilution with the respective BGE using various dilution factors (undiluted, fivefold and tenfold). The solutions were spiked at 0.2 µg/ml with the following 12 compounds: Gly, AMPA, *N*-acetyl-glyphosate (NAGly), fosetyl, ethephon, HEPA, glufosinate, 3-methylphosphinicopropionic acid (MPPA), *N*-acetyl-glufosinate (NAGlu), chlorate, perchlorate and phosphonate (also 'phosphonic acid', HPO_3_^2−^). The respective IL-ISs were added at 0.2 µg/ml. These solutions were injected repeatedly (*n* = 10, except Swiss chard extract undiluted *n* = 5), at this stage without using a focusing buffer (was introduced and studied at a later step). Swiss chard was chosen as an exemplary vegetable matrix, due to its high natural content of anionic compounds, e.g. nitrate and chloride [38], simulating a quasi worst-case matrix.

Special focus was placed on ensuring the electromigrational separation of phosphonate from compounds interfering with its measurement, i.e. fosetyl and phosphate (PO_4_^3−^), the latter being a natural matrix component. Furthermore, phosphonate was separated in standard solutions from fosetyl and ethephon as the latter two compounds (and their IL-ISs) tend to degrade to phosphonate in solution to some extent [9].

### Improvement of the injection conditions

#### Sample stacking using focusing buffers

Sample extracts along with focusing buffers of different concentrations were injected using several injection settings (pressure and duration) for each. The pressure applied and time period this pressure is maintained define the injection volume in nl (Supplementary Table S5). Both parameters were varied for the focusing buffer solutions and the sample extracts, with the injection of the sample extract being bracketed by injections of focusing buffer. In the present case, the focusing buffer solution was injected at (i) 50 pounds per square inch for seconds (psi*s) and (ii) 150 psi*s, and the sample extract at (iii) 200 psi*s and (iv) 400 psi*s (Supplementary Fig. S1). For the focusing buffer, the same injection settings were used in both injections. The aqueous focusing buffer contained NH_4_Ac (at pH 6.7), and its concentration was varied as follows: 0.1 mM, 1 mM, 2 mM, 5 mM, 10 mM, 25 mM, and 50 mM, with 0.1–2 mM being only evaluated for AMPA and glufosinate. In this experiment, QuPPe extracts of Swiss chard previously spiked at 0.2 µg/ml (with Gly, AMPA, NAGly, fosetyl, ethephon, HEPA, glufosinate, MPPA, NAGlu) were fivefold diluted with BGE and injected in triplicate (*n* = 3) for each experiment setup. In total, 16 different experiment setups (combinations of conditions) were checked.

#### Impact of injection settings on variability

Here, it was examined whether different combinations of pressure and pressure application time influenced the repeatability of the measured signals, expressed as relative standard deviation (RSD). For this purpose, the product of pressure × time was kept constant at 200 psi*s, with the pressure being varied between 2 and 20 psi (i.e. 138–1380 mbar). Ten injections of a standard mix solution at 0.2 µg/ml prepared in BGE (with Gly, AMPA, NAGly, fosetyl, ethephon, HEPA, glufosinate, MPPA, NAGlu, chlorate, perchlorate, phosphonate and the respective IL-ISs) were performed with each of the following settings: 2 psi (i.e. 138 mbar) for 100 s, 5 psi (i.e. 345 mbar) for 40 s, 10 psi (i.e. 689 mbar) for 20 s and 20 psi (i.e. 1380 mbar) for 10 s.

### Investigations of matrix effects

Matrix effects were determined by alternate injections of standard solutions containing the analytes of interest at equal concentrations, either in pure solvent (i.e. BGE) or in QuPPe extracts of a blank matrix. Matrix effects were calculated using Eq. (1) [39]:

1 $$ME\left(\%\right)=\left(\frac BA-1\right)\times100$$

where ME is matrix effect in %, with ‘0%’ indicating no matrix effect, and ‘ − 100%’ indicating total suppression. ME values < 0% indicate signal suppression, and ME values > 0% indicate signal enhancement. *A* is the signal intensity or peak area of the analyte in solvent or BGE, and *B* is the signal intensity or peak area of the analyte in matrix extract.

#### Matrix effects in undiluted sample extracts

A blank extract of Swiss chard was prepared from 10 g of cryogenically milled homogenate using the QuPPe-PO procedure, which corresponds to a matrix concentration in solvent of 0.5 g/ml [9]. The sample extracts (undiluted) were spiked at 0.2 µg/ml with both the following substances and their corresponding IL-ISs: Gly, AMPA, NAGly, HEPA, glufosinate, MPPA, NAGlu and chlorate. IL-ISs, which are well known to effectively compensate for matrix effects and other sources of error, were used to investigate whether they could also correct for the observed matrix effects in the present procedure [34].

#### Matrix effects in fivefold diluted sample extracts

Blank extracts of several matrices (liver, kidney, milk, rice, lemon, cucumber, Swiss chard and strawberry) were prepared from 10 g of cryogenically milled homogenate (exception: 5 g of rice homogenate) using either the QuPPe-PO [9] or the QuPPe-AO [36] procedure. The sample extracts were fivefold diluted with BGE and spiked at 0.2 µg/ml with the following 12 substances and their corresponding IL-ISs for correction (see Supplementary Table S2): Gly, AMPA, NAGly, fosetyl, ethephon, HEPA, glufosinate, MPPA, NAGlu, chlorate, perchlorate and phosphonate. This corresponds to a matrix concentration in solvent of 0.1 g/ml in the case of 10 g sample weight and of 0.05 g/ml in the case of 5 g sample weight (rice).

#### Impact of matrix on migration time shifts and peak shapes

Peak fronting and migration time shifts were examined by spiking chlorate and perchlorate at 0.2 µg/ml (without IL-IS) in undiluted and diluted (fivefold, tenfold and 20-fold) Swiss chard extracts.

### Method validation

Due to the contamination of standard substances (e.g. of fosetyl and fosetyl-D_5_ with phosphonate [9, 13]), method validation was carried out with two groups of analytes. The nine analytes of the first group (Gly, AMPA, NAGly, ethephon, HEPA, fosetyl, glufosinate, MPPA, NAGlu) were validated in strawberry, soybean, milk and liver following the protocol of an interlaboratory validation study carried out by the EURL-SRM. In brief, the protocol thoroughly describes the QuPPe procedure, relevant steps for method validation, applied spiking levels, as well as pipetting schemes for working solutions and extracts for measurement [40]. The second group of analytes (phosphonate, chlorate, perchlorate, trifluoroacetic acid (TFA), bromide) was validated in lemon and milk. The sample homogenates were spiked in quintuplicate (*n* = 5) before extraction at the respective levels and extracted following the QuPPe procedure for PO [9] or AO [36] commodities. The average recoveries were calculated using matrix-matched bracketing calibration at 60% and 120% of the validated level. In addition, matrix-based calibration standards were injected in cucumber extracts. To compensate for matrix effects and other sources of bias, IL-ISs were added between 0.1 and 0.4 mg/kg in 10 g sample (Supplementary Table S2). The provisions of the European guidance document SANTE/11312/2021 v2, i.e. average recoveries between 80 and 120% and RSD < 20%, were used to judge the performance in validation [41]. For glufosinate, injection conditions had to be developed individually, which entailed skipping the focusing buffer and diluting with BGE (see 'Validation of the CE-MS/MS method'). Individual conditions were also tested for AMPA, but generic conditions were finally used in validation (see 'Validation of the CE-MS/MS method'). The extracts were fivefold diluted with MeOH_FA_/H_2_O (7/3, v/v) (except for glufosinate, which required different conditions; see 'Validation of the CE-MS/MS method') and measured with CE-MS/MS.

## Results and discussion

### Effect of the BGE solution on the signal intensities and separation of analytes in CE-MS/MS

**Fig. 1:**
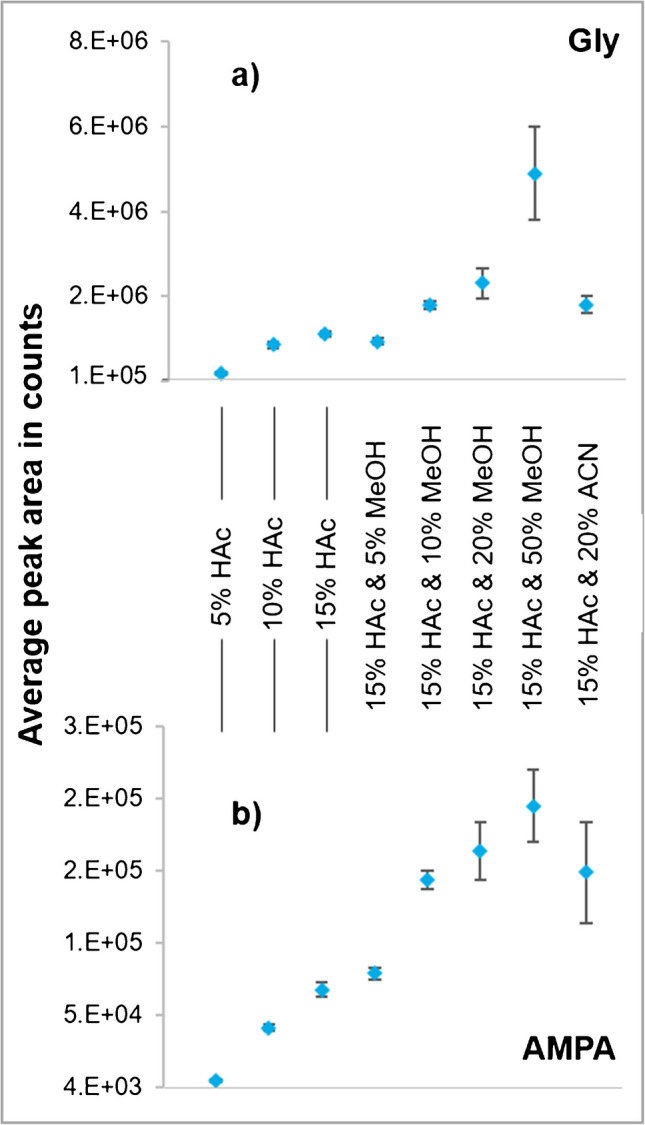
Average CE-MS/MS peak areas (*n* = 10) of Gly (**a**) and its main metabolite AMPA (**b**) at a concentration of 0.2 µg/ml in fivefold diluted QuPPe extracts of Swiss chard using different compositions of BGE as shown in the graph [36]. The dilutions prior to injection were conducted using the respective BGE. A further comparison of the different dilutions of four BGE compositions (5% HAc, 15% HAc, 15% HAc and 10% MeOH, 15% HAc and 20% MeOH) is shown in Supplementary Fig. S2

Different MS-compatible buffer salts or ionic components were tested for utilization in the BGE, but unfortunately resulted in poor or even no signals (i.e., at pH > 7 with NH_3_, with FA and especially NH_4_Ac; Supplementary Fig. S3). In contrast, first signals were achieved under acidic conditions using HAc. Furthermore, increasing the HAc content in the BGE from 5 to 15% improved the signal abundances of Gly and AMPA (Fig. 1a, b, left side). In contrast, a BGE with only 1% HAc resulted in very poor signals (data not shown). Hence, further investigated compositions with organic solvent were based on an HAc content of 15%. Keeping the HAc content at 15%, the addition of MeOH in the BGE further increased the signal intensities (Fig. 1a, b, central part). While the highest peak area was obtained with 50% MeOH in BGE, the use of such a high MeOH content was accompanied by stronger variations in the peak areas in replicate injections (*n* = 10, RSD 22%) (Fig. 1a, b). Similarly disadvantageous results were obtained with 15% HAc and 20% MeOH or ACN (RSD 22%), especially for AMPA (Fig. 1b, right side). Fortunately, RSDs were negligible at 5% and 10% MeOH in BGE (Fig. 1a, b).

Since run-to-run variations in the signal intensities can be corrected by the IL-IS, which is generally used in the QuPPe extraction procedure (see also 'Injection of the sample extracts'), a BGE mixture containing 15% HAc and 20% MeOH in water was considered best in terms of good sensitivity paired with only a moderate RSD at this point.

**Fig. 2:**
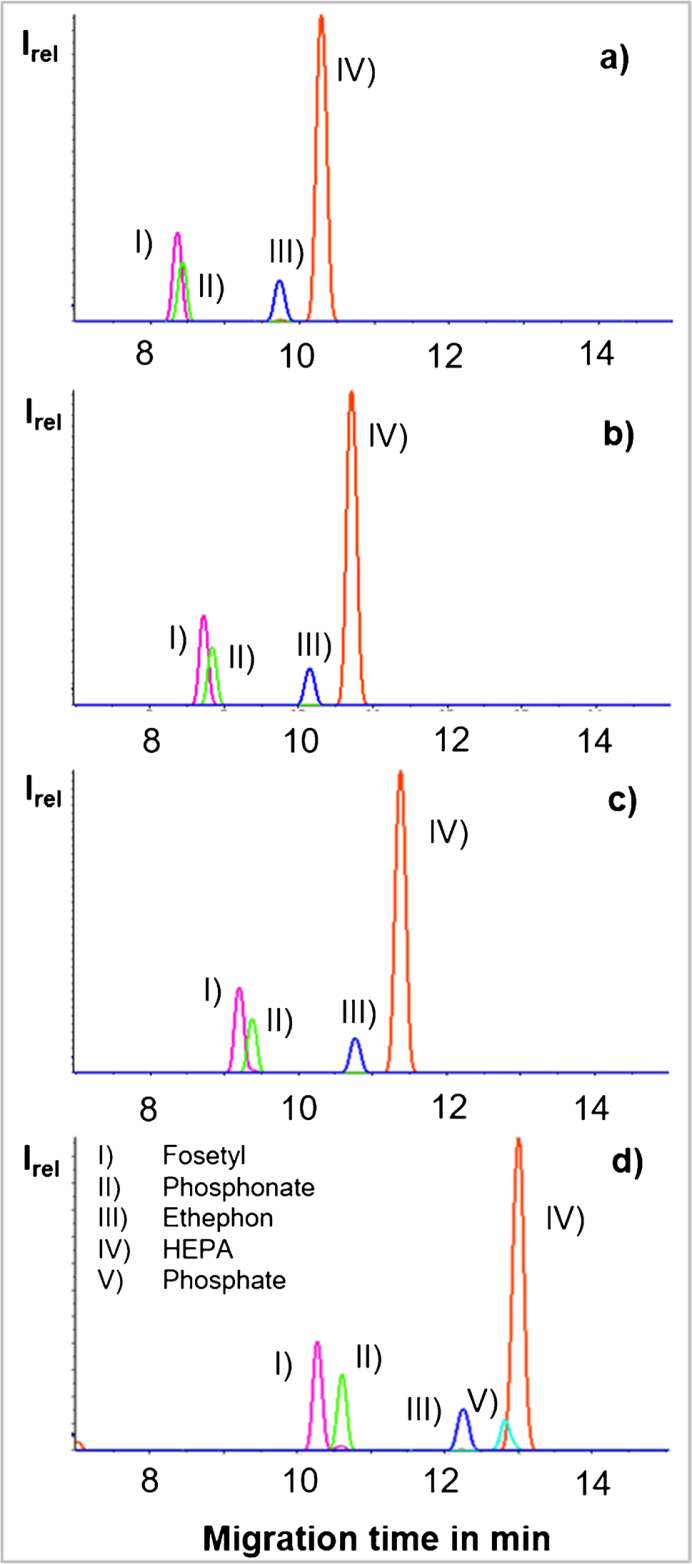
Exemplary CE-MS/MS electropherograms of several analytes (target mass transitions) as well as of phosphate using as BGEs 15% aqueous HAc solutions containing 0% MeOH (**a**), 5% MeOH (**b**), 10% MeOH (**c**) or 20% MeOH (**d**) (final conditions), and the separation of phosphonate from fosetyl (critical pair), as well as from ethephon and HEPA (shown in **a**–**c**; phosphate not measured), and separation of phosphonate from phosphate (critical pair, shown in **d**). The solutions were spiked with the analytes at a concentration of 0.2 µg/ml and were prepared in the respective BGE. The migration time windows show the analytes whose separation was considered most important

Moreover, the use of MeOH in BGE not only improved the signal abundance, but also strongly increased the migration times of the analytes (Fig. 2), due to a thereby increased viscosity of the BGE and changes in the electro-osmotic velocity [15, 17]. Therefore, increasing the MeOH content from 5% over 10 to 20% (Fig. 2) steadily improved the separation of fosetyl and phosphonate, and both analytes were baseline separated with the highest MeOH content of 20% (Fig. 2d). The separation of the two analytes was aspired in order to avoid mass spectrometric interferences; see also ‘Experimental’ [9]. These conditions were used subsequently, despite the partial co-migration of HEPA with phosphate, which caused ion suppressions on HEPA in matrix extracts (see section ‘Investigation of matrix effects’). Under these final conditions, all analytes eluted between ~ 8 and 16 min (Supplementary Fig. S4).

### Investigation of different injection conditions in CE-MS/MS

#### Sample stacking using focusing buffers

After selecting an appropriate BGE composition, the injection conditions were further investigated by selecting the applied pressure and pressure application time during the injection of both the sample extract and the focusing buffer (i.e. injected volume, as the combination of applied pressure and its duration defines the injected volume in CE). For both the focusing buffer and the sample, two different settings were chosen, i.e. (i) a low (50 psi*s) or (ii) a threefold higher buffer injection volume (150 psi*s), and (iii) low sample extract injection volumes (200 psi*s, corresponding to ~ 38 nl; Supplementary Table S5) and (iv) twofold higher (400 psi*s, corresponding to ~ 76 nl). In addition, the concentration of the focusing buffer was varied as well (5 mM, 10 mM, 25 mM and 50 mM) (Supplementary Fig. S1).

Injections of sample volumes ≥ 200 psi*s resulted in considerable peak broadening and no sensitivity gains (data not shown). While utilizing a buffer in BGE aimed to control the pH value (but resulted in signal loss), its use during injection aimed at keeping the analyte band relatively narrow even when injecting relatively large sample volumes [42]. For this purpose, the sample extract was injected between two buffer volumes (Supplementary Table S3). Specifically, larger sample volumes can increase the sensitivity but may necessitate a focusing step to maintain narrow bands of the analytes [42]. In this manner, Stuke et al. [32] have successfully used a 50 mM NH_4_Ac buffer for focusing the migration bands of TFA and difluoroacetic acid (DFA).

**Fig. 3:**
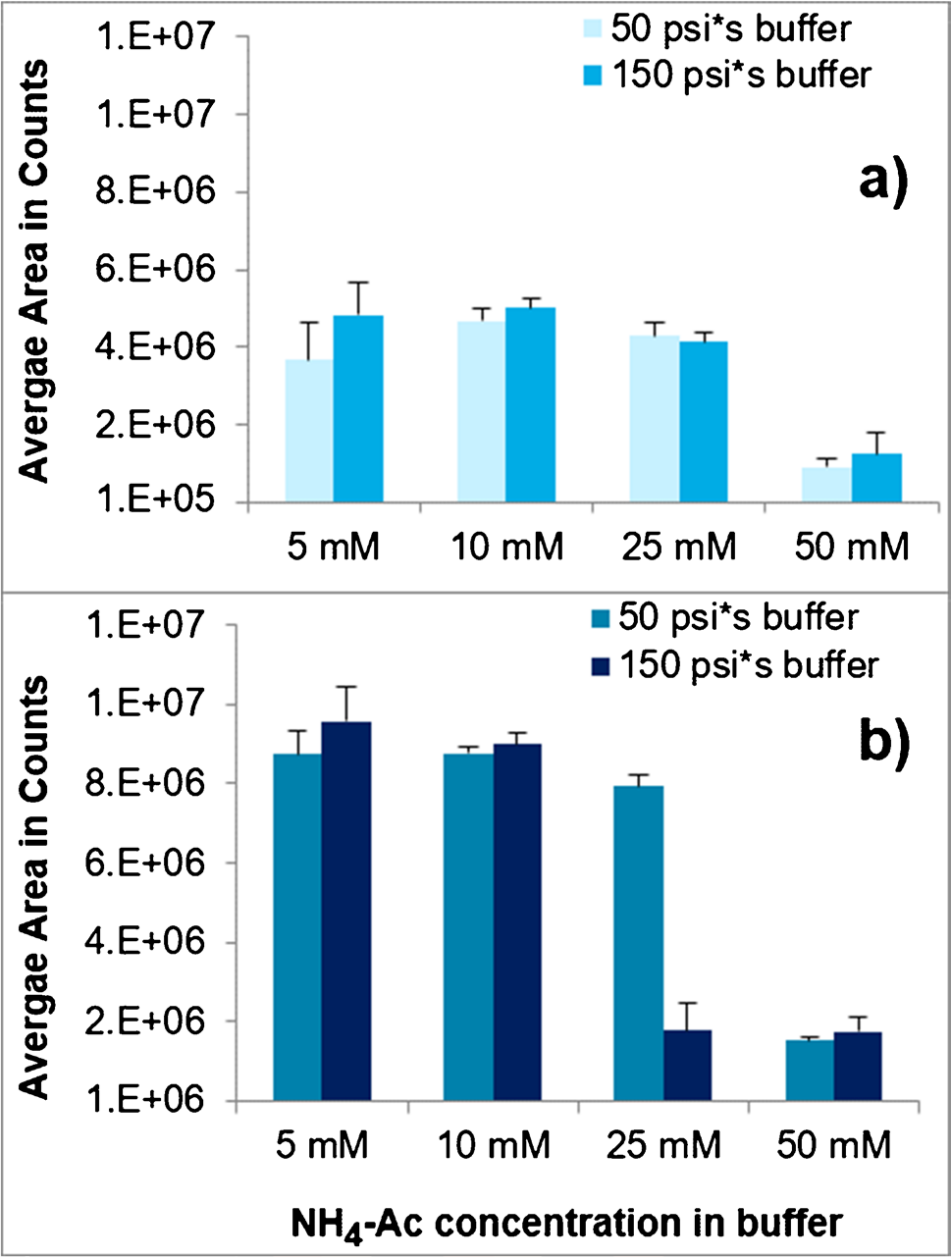
Comparison of average CE-MS/MS peak areas of Gly injected at a concentration of 0.2 µg/ml (*n* = 3) at varying concentrations of the focusing buffer solution (5 mM, 10 mM, 25 mM or 50 mM NH_4_Ac in water) and the varying settings as regards the pressure and duration of injection (both before and after the sample extract): (i) 50 psi*s (5 psi for 10 s) or (ii) 150 psi*s (10 psi for 15 s). On the top (**a**), the sample extract was injected with (iii) 200 psi*s (10 psi for 20 s), and on the bottom (**b**), with (iv) 400 psi*s (10 psi for 40 s). The solutions were prepared by fivefold diluting QuPPe extracts of Swiss chard with BGE and spiking them with the analytes at 0.2 µg/ml

An NH_4_Ac concentration of 5 mM, which was used as a starting point, resulted in an average peak area of Gly of 3.7 × 10^6^ counts when injecting (i) 50 psi*s buffer and (iii) 200 psi*s sample extract (Fig. 3a). Slightly higher peak areas were obtained with 10 mM (4.7 × 10^6^ counts) and 25 mM (4.3 × 10^6^ counts) NH_4_Ac (Fig. 3a). However, at 50 mM in the focusing buffer, NH_4_Ac caused a sharp drop of the signal of Gly by ~ 75% (Fig. 3a). This loss in sensitivity was independent of the volume of buffer (i or ii) or sample (iii or iv) injected (Fig. 3a, b). A similarly strong loss of signal (− 75%) was also observed at 25 mM NH_4_Ac when injecting larger volumes of sample (iv) (400 psi*s) and focusing buffer (ii) (150 psi*s) (Fig. 3b and Supplementary Fig. S6).

Doubling the injected sample volume (from (iii) 200 psi*s to (iv) 400 psi*s exemplarily shown for Gly; Fig. 3a, b) roughly doubled the peak areas of virtually all investigated analytes when NH_4_Ac was added at 5 mM or 10 mM to the buffer solution. However, injecting larger sample volumes caused peak broadening, which could not be overcome with the help of the focusing buffer (Supplementary Figs. S5 and S6a). In fact, peak broadening was observed with > 5 mM NH_4_Ac (Supplementary Fig. S5b) in the focusing buffer or by applying 150 psi*s injection volume of buffer (ii) (Supplementary Figs. S5c and S6a) instead of 50 psi*s (i). For the final conditions, it was therefore decided to combine (iii) 200 psi*s for the sample extracts and (i) 50 psi*s for the buffer solution at a concentration of 5 mM NH_4_Ac.

Still, AMPA (Supplementary Fig. S7a, b) and glufosinate were exceptions since signal loss was observed when injecting a buffer (of any concentration). In contrast to the other analytes, signal loss was observed for AMPA and glufosinate at any buffer concentration (Supplementary Fig. S8). Due to the importance of AMPA and glufosinate, it was therefore decided to run the analysis of these two analytes separately without the use of a focusing buffer (i.e. without NH_4_Ac).

#### Injection of the sample extracts

**Fig. 4:**
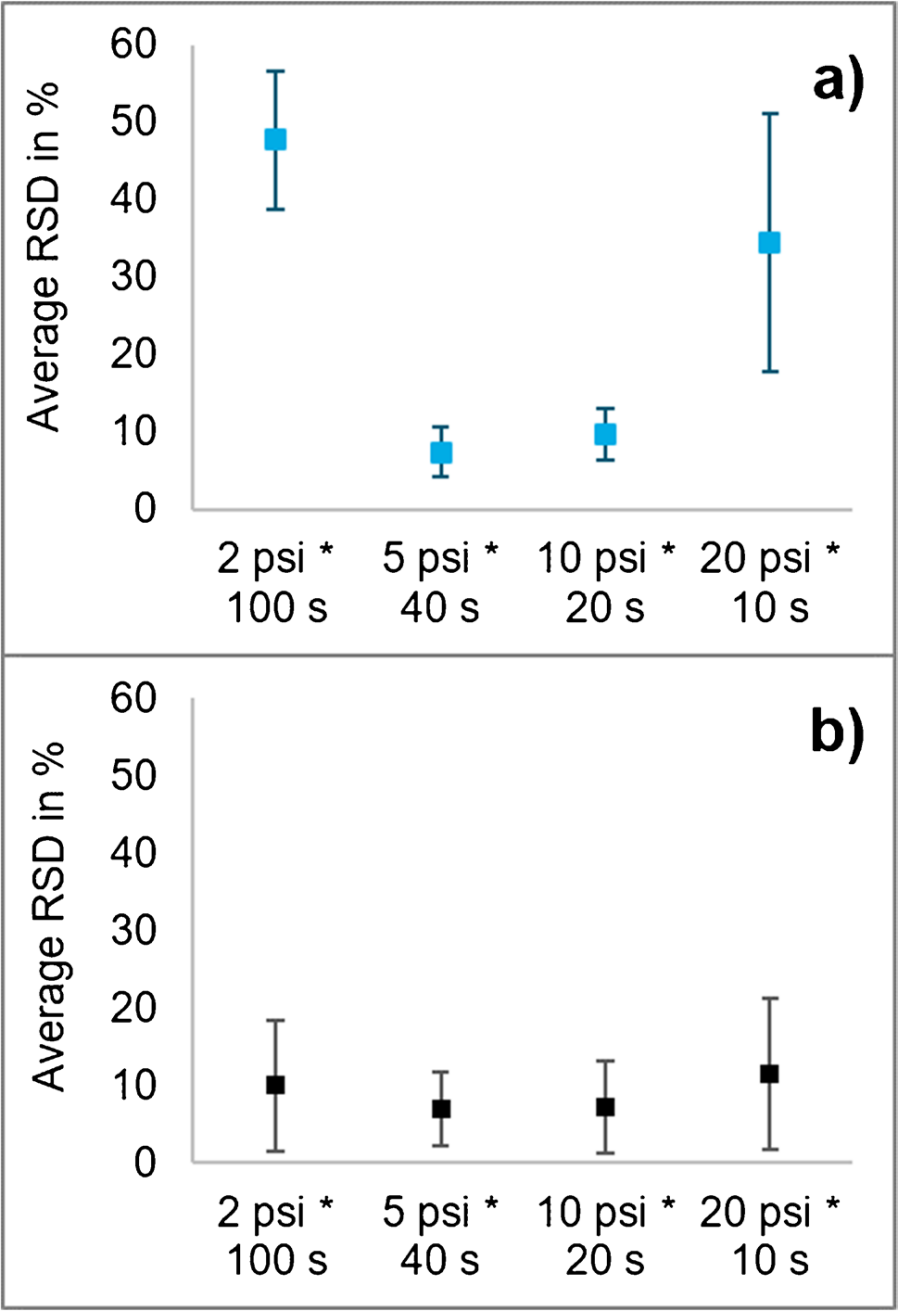
Average RSDs of 12 tested compounds (*n* = 12) spiked with the analytes at 0.2 µg/ml in BGE derived from 10 replicate injections at each injection condition. Four different combinations of injection pressure and time were used, all resulting into 200 psi*s: 2 psi*100 s, 5 psi*40 s, 10 psi*20 s and 20 psi*10 s. On the top (**a**), without the use of IL-IS, and on the bottom (**b**), using IL-IS

When applying different combinations of pressure and pressure application time at a constant product of pressure × time of 200 psi*s (with the pressure being varied between 2 and 20 psi, i.e. 138–1380 mbar), the RSDs of the 12 tested compounds of a standard mixture in BGE ranged between 4 and 17% when using 5 psi (i.e. 345 mbar for 40 s) or 10 psi (i.e. 689 mbar for 20 s) (Fig. 4a). In contrast, RSDs were much higher at lower or higher pressure (≥ 30% at 2 psi (i.e. 138 mbar for 100 s) and between 11 and 65% at 20 psi (1380 mbar for 10 s)) (Fig. 4a). Nevertheless, the average RSD of the analytes was generally ≤ 10% after correction with (the co-injected) IL-IS (Fig. 4b). To save analysis time and keep variability low, it was finally decided to apply 10 psi (i.e. 689 mbar) for 20 s (10 psi*20 s) for the injection of sample extracts.

### Investigation of matrix effects

Once a suitable composition of the BGE (15% HAc and 20% MeOH in water), and the conditions of the focusing buffer (5 mM NH_4_Ac and 50 psi*s injection volume, except for AMPA and glufosinate; see 'Effect of the BGE solution on the signal intensities and separation of analytes in CE-MS/MS'), and the sample injection (10 psi for 20 s) were established, the potential matrix effects were studied in several commodities of plant and animal origin.

#### Matrix effects in undiluted sample extracts

**Fig. 5:**
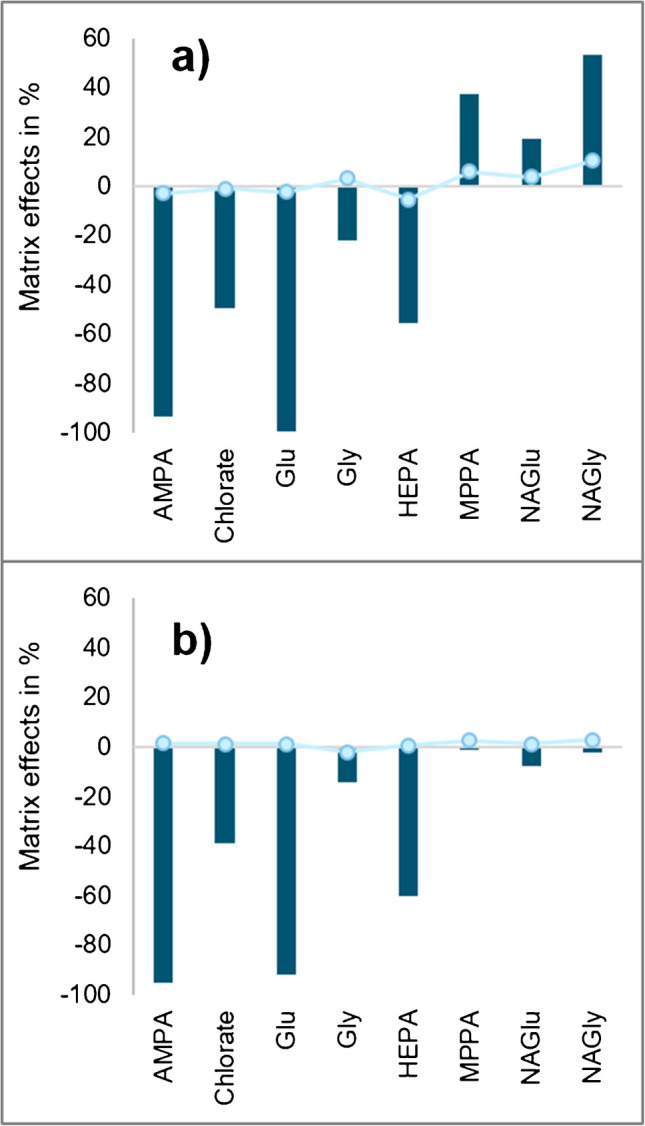
Selected examples of matrix effects observed in CE-MS/MS for Gly, Glu and their metabolites (AMPA, NAGly, MPPA and NAGlu), as well as of chlorate in undiluted Swiss chard extracts (**a**) and in Swiss chard extracts following fivefold dilution with BGE (**b**). The dark blue bars show the matrix effects based on peak areas. ‘0%’ means total suppression; matrix effect values > 0 means signal enhancement. The light blue dots show how the respective area ratios between analyte signal and IL-IS signal deviate from those in solvent. The ME formula (Eq. 1) was applied for the calculations

During the investigation of the BGE composition, it was observed that CE-MS/MS signal intensities, especially of AMPA and glufosinate (Glu), were smaller in Swiss chard extracts than in BGE/solvent at almost every BGE composition tested (for AMPA, see 'Effect of the BGE solution on the signal intensities and separation of the analytes in CE-MS/MS' and Supplementary Fig. S2). The presence of matrix furthermore resulted in a moderate suppression (chlorate and HEPA) and a moderate signal enhancement (MPPA and NAGly; Fig. 5a), whereas matrix effects on Gly and NAGlu were negligible to moderate (-22% and +19%, Fig. 5a) according to the SANTE guidelines (negligible = within ± 20%) [41]. However, the reported matrix effects were successfully compensated by the use of IL-IS in each case (Fig. 5). The used IL-ISs in this study were labelled with ^2^H, ^13^C, ^15^N or ^18^O. Being (i) chemically identical to their corresponding analytes, IL-ISs (ii) exhibit the same behaviour during analysis, thus (iii) co-migrating during CE separation and (iv) exhibiting the same ionization efficiency in the ion source [34]. In order to be distinguished from native analytes by MS, a mass difference of *m*/*z* values of at least 3 between native and IL-IS compounds is usually adequate [34]. The effectiveness of the employed IL-ISs in compensating for matrix effects was demonstrated (Fig. 5).

#### Matrix effects after a fivefold dilution of sample extracts (consideration without IL-IS)

The signal suppressions observed in undiluted extracts could be partly reduced by diluting the sample solutions. A fivefold dilution of Swiss chard extracts was effective and shifted the matrix effects to the acceptable range of ± 20% in the case of MPPA, Gly and NAGly (Fig. 5b). By contrast, signal suppressions of AMPA, glufosinate and HEPA remained ≤ -60% (Fig. 5b). Coherently, the signals of AMPA in diluted matrix extracts were at least 70% smaller than in pure BGE (Supplementary Fig. S1b).

**Fig. 6:**
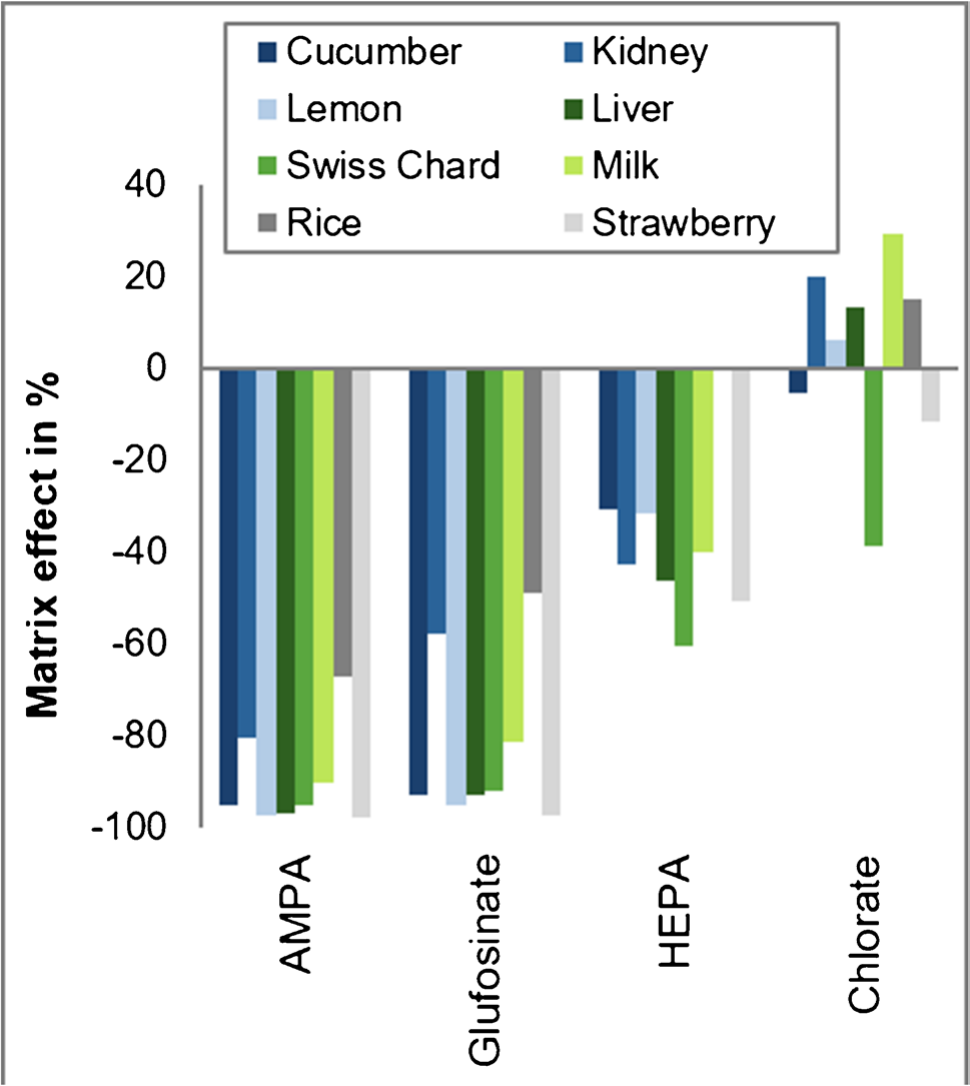
Matrix effects on exemplary compounds (AMPA, glufosinate, HEPA and chlorate, which were most heavily affected by ion suppression) observed during CE-MS/MS analysis when injecting fivefold diluted extracts of different matrices of plant and animal origin spiked with the analytes at 0.2 µg/ml [36]; ‘0%’ means no matrix effect, ‘ − 100%’ means total suppressions, and matrix effect values > 0% mean signal enhancement

AMPA, which is also often affected by strong matrix effects in LC-MS/MS [13], was also the most affected analyte in CE-MS/MS, experiencing the strongest signal suppression in all 7 matrices (between -67% in rice and even -98% in strawberry; Fig. 6), followed by glufosinate (between -49% in rice and -97% in strawberry). HEPA and chlorate, which experienced the strongest signal suppression in Swiss chard extracts (Fig. 5), showed rather moderate or minor signal suppressions in the other matrices examined in this study (Fig. 6). Comparably low matrix effects were observed for the other 8 tested compounds ranging between -28% (maximum signal suppression for NAGlu in lemon) and +37% (maximum signal enhancement for MPPA in milk) (Supplementary Fig. S9).

Under the acidic conditions of the BGE, not every compound was fully deprotonated in the equilibrium established under the given pH, especially AMPA and glufosinate (Supplementary Table S6) [43]. For compounds having a net charge close to zero or even a positive net charge, CE separation (in the anion mode) becomes inefficient as analytes are moved through the capillary mainly (or only) by the applied supporting pressure [43]. However, a change of pH conditions in the BGE towards higher pH values compromised the sensitivity (see 'Effect of the BGE solution on the signal intensities and separation of the analytes in CE-MS/MS') and was thus not further investigated.

Since ultra-low flow rates are used in CE (similarly to nano-LC), fewer matrix co-extractives reach the ion source and less competition during ionization can be expected. CE therefore tends to be less affected by signal suppression [18, 44]. When using IC-MS/MS and LC-MS/MS, AMPA and glufosinate were also more affected by matrix effects in fivefold diluted sample extracts than other compounds [13]. Overall, matrix effects in CE were moderate and, for most compounds, less problematic than in IC- and LC-MS/MS (see 'Matrix effects in undiluted sample extracts') [13]. Unfortunately, signal suppressions of AMPA and glufosinate were even more pronounced in CE-MS/MS compared to the other two techniques. The hope that CE-MS/MS would be a viable alternative for the notoriously problematic compound AMPA did not materialize.

#### Matrix impact on migration times and peak shapes

**Fig. 7:**
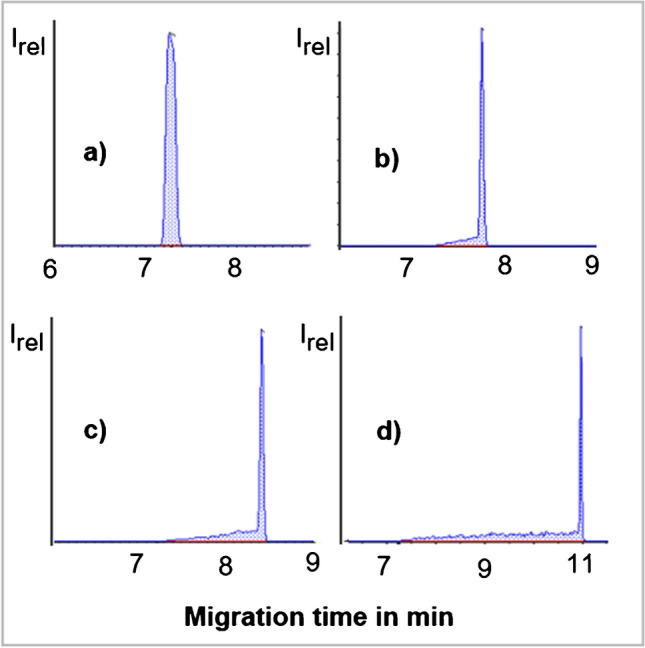
Shifts of CE-MS/MS migration time and peak shape (fronting) of chlorate (at 0.2 µg/ml) in Swiss chard extracts when injected following dilution at different rates: 20-fold (**a**), tenfold (**b**), fivefold (**c**) and undiluted (**d**). Corresponding peak shapes of perchlorate are shown in Supplementary Fig. S10

The presence of matrix compounds caused migration time shifts and peak fronting in the case of chlorate and perchlorate (Fig. 7 and Supplementary Fig. S10). Under the applied conditions, chlorate and perchlorate had similar migration times as chloride and nitrate, which are highly abundant natural matrix compounds in Swiss chard and which were co-extracted with the QuPPe method [38]. It is conceivable that especially during the initial stages of the CE run, an excess amount of matrix components co-migrated with chlorate and perchlorate hindering their proper preconcentration within the CE capillary, thus resulting in the observed altered peak shapes. In a separate experiment, it could be shown that the anions chloride and nitrate, when added to reagent blanks at high concentrations (i.e. > 250 µg/ml each), cause a similar broadening to the peaks of chlorate and perchlorate (not shown). By diluting the extracts prior to injection, peak fronting was reduced but was still very strong at a fivefold dilution and still recognizable at a tenfold dilution. Fortunately, chlorate and perchlorate show a very sensitive detection and can tolerate high dilution factors.

### Overall insights concerning matrix effects on polar pesticides in CE-MS/MS

Signal suppressions, migration time shifts and peak distortions noted in undiluted matrix extracts could be reduced effectively in most cases by a fivefold dilution of QuPPe extracts (Supplementary Figs. S2b, S7 and S10). Remarkable exceptions were AMPA (Supplementary Fig. S2b) and glufosinate. Since dilution factors > 5 were disadvantageous for Gly (Supplementary Fig. S2a) and other analytes, a fivefold dilution was considered the best compromise for most applications, similarly to IC-MS/MS [13]. However, sample matrices containing high amounts of nitrate or chloride had to be diluted and re-analysed for chlorate and perchlorate. Furthermore, it was advantageous for the analysis of AMPA and glufosinate to further dilute sample extracts, when the residue levels were high enough for their detection. Irrespective of this, all observed CE-MS/MS signal deviations could be corrected by the use of IL-ISs.

### Validation of the CE-MS/MS method

The previous experiments indicated that a method employing (1) a concentration of 15% HAc and 20% MeOH in the BGE, (2) injection of 200 psi*s of fivefold diluted extracts (dilution with MeOH_FA_/H_2_O (7/3, v/v)) and (3) 50 psi*s for injection of the buffer solution at a concentration of 5 mM NH_4_Ac (except for AMPA and glufosinate; see 'Experimental') resulted in good performance for most analytes.

Generally, the IL-IS-corrected recovery rates of the spiked pesticides and using matrix-matched calibration met the requirements regarding recovery in pesticide residue analysis as stated in the European guidance document SANTE/11312/2021 v2 [41] (Tables 1 and 2). However, limitations were observed in soybeans, where due to the strong suppression, the sensitivity of the CE-MS/MS method was too poor for the determination of AMPA and glufosinate at a spiking level of 0.1 mg/kg and 0.06 mg/kg, respectively. Also in soybeans, the RSDs achieved for Gly and MPPA were > 20% despite the IL-IS correction at this spiking level (0.1 mg/kg Gly and 0.04 mg/kg MPPA, exact spiking levels in Table 1). In addition, validation of AMPA in strawberry and TFA in lemon failed at the lowest spiking level (0.05 mg/kg for AMPA and 0.025 mg/kg for TFA), in contrast to milk where validation at 0.05 mg/kg AMPA and 0.0025 mg/kg TFA was successful. In these cases, the present CE-MS/MS method was less suitable than IC-MS/MS or LC-MS/MS methods, which were performing well at these spiking levels and matrices [13]. Except for Gly in strawberry and perchlorate in lemon (average recovery rate > 120%), as well as for perchlorate in milk and fosetyl in soybean (RSD > 20%), the CE-MS/MS method also performed well using a generic and thus more routine-compatible matrix-based calibration of the 14 analytes including Gly (see also data on matrix-based calibration on cucumber extracts in Supplementary Tables S7 and S8). With the presented lowest successfully validated levels for Gly being equal or below the currently applicable MRLs (strawberry 0.1 mg/kg, milk 0.05 mg/kg, liver 0.2 mg/kg, soybeans 20 mg/kg), we believe that the developed method is well suitable for effectively controlling the compliance of Gly residues in commodities beyond those exemplarily tested in this study [45]. The presented achieved LSVLs can be considered as the limits of quantitation (LOQs) of the method.

**Table 1:** Average recovery rates (*n* = 5) and RSDs in the CE-MS/MS analysis of highly polar anionic pesticides in strawberry, milk, liver and soybean at the respective lowest successfully validated level. Results shown were calculated based on 2-point bracketing matrix-matched calibration with the use of IL-IS. All extracts were fivefold diluted with MeOH_FA_/H_2_O (7/3, v/v), except for glufosinate, which was diluted with BGE

Analyte (target/quantifier mass transition; see Supplementary Table 4)	Spiking level (mg/kg)	Strawberry		Milk		Liver		Spiking level (mg/kg)	Soybean	
		Average recovery (%)	RSD (%)	Average recovery (%)	RSD (%)	Average recovery (%)	RSD (%)		Average recovery (%)	RSD (%)
Gly	0.05	97	13	106	5	91	12	0.1	87	22^a^
AMPA	0.05	97	36^a^	92	6	99	11	0.1	n.d.^b^	–
NAGly	0.05	97	2	102	4	99	12	0.1	101	4
Glufosinate^c^	0.06	100	17	94	14	80	5	0.06	n.d.^b^	–
MPPA	0.02	103	5	107	5	93	9	0.04	83	27^a^
NAGlu	0.02	98	9	89	17	107	25^a^	0.04	86	16
Ethephon	0.01	96	3	95	6	123^d^	13	0.02	106	20
HEPA	0.02	101	4	111	10	n.a.^e^	–	0.04	96	9
Fosetyl	0.01	98	8	108	10	98	8	0.02	99	19

^a^Required performance criteria as regards repeatability (RSD ≤ 20%) not fulfilled in these cases (due to strong signal suppression and poor signal intensity)

^b^No peak detected at the envisaged level

^c^Results for glufosinate obtained using cutomized settings entailing dilution in BGE and injection without bracketing with focusing buffer

^d^Average recovery outside the range (80 - 120%) where correction for bias may be omitted

^e^A proper validation of HEPA in liver at this spiking level was not feasible because of natural occurrence of HEPA in liver [37]

*n.a.* not analysed, *n.d.* not detected

**Table 2:** Average recovery rates (*n*=5) and relative standard deviations (RSDs) in lemon and milk at the respective lowest successfully validated level. Results shown were calculated based on 2-point bracketing matrix-matched calibration with the use of isotope labelled internal standards (IL-IS) (except bromide). All extracts were 5-fold diluted with MeOH_FA_/H_2_O 7/3

Analyte (target/quantifier mass transition; see Supplementary Table 4)	Spiking level (mg/kg)	Lemon		Spiking level (mg/kg)	Milk	
		Average recovery (%)	RSD (%)		Average recovery (%)	RSD (%)
Bromide	5	97	11	5	97	9
Chlorate	0.03	92	12	0.03	92	7
Perchlorate	0.01	99	11	0.02	101	20
Phosphonate	0.05	105	5	0.05	107	7
TFA	0.05	87	4	0.025	99	13

Compared to other CE-MS/MS methods [14, 31, 33], the analysis of QuPPe sample extracts with the presented CE-MS/MS method was less sensitive, which was mainly owed to the minimal sample clean-up, the use of more complex sample matrices and the instrumental designs (e.g. sheath-assisted interface achieved better sensitivities [14, 31]), and that no enrichment step was implemented. For instance, Wimmer et al. [14] reported very low limits of detection (LODs, which was defined as a signal-to-noise ratio = 3) of Gly in beer (6.2 µg/l) and AMPA (24.4 µg/l) and even lower LODs in water (without matrix). Furthermore, Graf et al. [31] achieved remarkably low LODs of 25 ng/l with a sophisticated “two-dimensional column coupled isotachophoresis/CZE-MS” method in water. Also, Liu et al. [33] validated Gly, AMPA, glufosinate and MPPA at 0.01 mg/kg in baby food using the same sheathless interface after enrichment by titanium dioxide–coated core–shell silica microsphere–based (CSMS@TiO_2_) solid-phase extraction. Despite the lower sensitivity, the present CE-MS/MS method was found to be well suited for routine analysis as it allowed to control MRLs of Gly and related analytes in very complex sample matrices with minimal sample clean-up (QuPPe). The method allows for a straightforward determination of the target analytes without cumbersome and complicated derivatizations or enrichments of single analytes. The measurement of extracts derived from the QuPPe method, which covers > 50 analytes, opens the way for applying the method in laboratories involved in routine controls of pesticide residues in food and awaiting publication as EN 18032 in 2025 [9, 46, 47].

## Conclusion

The experiments conducted in this study have shown that CE-MS/MS is suitable for the quantification of residues of several highly polar pesticides in complex food commodities following QuPPe extraction. Different factors influenced the separation and sensitivity and therefore had to be evaluated beforehand. Particularly, the composition of the background electrolyte (BGE) was crucial for both the sensitivity and the chromatographic resolution of analytes.

Once established, CE-MS/MS provided a better separation of Gly and other analytes from the matrix co-extractives compared to LC-MS/MS methods. Specifically, most of the targeted analytes including Gly showed only moderate ion suppression or enhancement that could be well compensated by extract dilution and IL-IS correction. However, the performance was worse in the case of AMPA and glufosinate. Hence, the analysis of AMPA and glufosinate was only successful at medium to high residue levels. After successful method validation for most of the tested matrix/spiking-level combinations, the inter-injection variability was higher compared to LC- and IC-MS/MS methods [9, 13]. However, also this disadvantage could be overcome through IL-IS correction, which again turned out to be essential for obtaining acceptable accuracy and precision by compensating deviations during extraction, injection and ionization (matrix effects).

One of the major benefits of CE-MS/MS is the use of ultra-low flow rates paired with an ultra-low sample load. These basic characteristics of CE are in favour of a good ionization yield in an ESI source and less instrument contamination, similar to nano-LC [6, 44]. Using online tools based on theoretical calculations [48], the sample injection volume of the presented method was estimated to be approximately 40 nl which is 10 to 100 times less than the volumes typically injected in LC- and IC-MS/MS. Considering these low injection volumes and the peak areas obtained, this is a clear confirmation of higher ionization yields in CE.

## Supplementary Information

Below is the link to the electronic supplementary material.

Supplementary Material 1: The online version contains supplementary material available at … (PDF 799 KB)

## Data Availability

The data presented in this study are available from the corresponding author on request.

## Declarations

### Source of biological material

Biological material from organic production (of plant and animal origin) was purchased at local supermarkets.
