# Supplementary material for: Analysis of several anionic polar pesticides in food of plant and animal origin using QuPPe extraction and CE-MS/MS determination
Source: Anal Bioanal Chem. 2025 Jul 21;417(21):4739–52. doi: 10.1007/s00216-025-05990-1 (PMC12367890; doi:10.1007/s00216-025-05990-1)
Supplement: Supplementary file 1 — Supplementary Material 1: The online version contains supplementary material available at … (PDF 799 KB) [file 216_2025_5990_MOESM1_ESM.pdf]

Supplementary Information for

Analysis of several anionic polar pesticides in food of plant and animal origin using QuPPe extraction and CE-MS/MS determination

In *Analytical and Bioanalytical Chemistry*

Ann-Kathrin Schäfer[1*], Walter Vetter[2] and Michelangelo Anastassiades[1]

[1]Chemisches und Veterinäruntersuchungsamt Stuttgart, Section for Residues and Contaminants, D-70736, Fellbach, Germany

[2]University of Hohenheim, Institute of Food Chemistry (170b), D-70599, Stuttgart, Germany

*Corresponding author

Email address: Ann-Kathrin.Schaefer@cvuas.bwl.de

Table S1: *List of abbreviations.*

Abbreviation	Definition
ACN	acetonitrile
AMPA	aminomethylphosphonic acid
AO	animal origin
BGE	background electrolyte
CE	capillary electrophoresis
CE-MS/MS	capillary electrophoresis with tandem mass spectrometry
CZE	capillary zone electrophoresis
dSPE	dispersive solid phase extraction
EDTA	ethylenediaminetetraacetic acid
EURL-SRM	European reference laboratory for pesticides requiring single residue methods
FA	formic acid
FMOC-Cl	fluorenylmethyloxycarbonyl chloride
GC	gas chromatography
GC-MS/MS	gas chromatography with tandem mass spectrometry
Gly	glyphosate
HAc	acetic acid
HEPA	2-hydroxyethylphosphonic acid
HCl	hydrochloric acid
HPLC	high performance liquid chromatography
IC	ion chromatography
IC-MS/MS	ion chromatography with tandem mass spectrometry
IL-IS	isotopically labelled-internal standard
LC	liquid chromatography
LC-MS/MS	liquid chromatography with tandem mass spectrometry
ME	matrix effect
MeOH	methanol
$MeOH_{FA}$	MeOH containing 1% formic acid
MPPA	3-methylphosphinicopropionic acid
MS/MS	tandem mass spectrometry
NAGlu	*N*-acetyl-glufosinate
NAGly	*N*-acetyl-glyphosate
NaOH	sodium hydroxide
NH_4Ac	ammonium acetate
ODS	octadecylsilane
PO	plant origin
QuPPe	quick polar pesticides (method)
RSD	relative standard deviation
TFA	trifluoroacetic acid
TOF-MS	time of flight mass spectrometry

Table S2: *Isotope-labelled internal standards (IL-ISs) used, concentration in spiking solutions, of which 100 µL were added prior to extraction in validation experiments, concentration in 10 g and 5 g sample portion and measured mass transitions in MS/MS, see also [1].*

**Compound**	**Concentration of spiking solutions in µg/mL**	**Concentration in 10 g sample portion in mg/kg**	**Concentration in 5 g sample portion in mg/kg**	**Mass transition**
**Glyphosate $^{13}C_2$ ^{15}N**	20	0.2	0.4	*m/z* 171 → 63
**AMPA ^{13}C ^{15}N**	40	0.4	0.8	*m/z* 112 → 63
***N*-Acetyl-Glyphosate $^{13}C_2$ ^{15}N**	20	0.2	0.4	*m/z* 213 → 63
**Fosetyl D_5**	20	0.2	0.4	*m/z* 114 → 82
**Ethephon D_4**	20	0.2	0.4	*m/z* 147 → 111
**HEPA D_4**	20	0.2	0.4	*m/z* 129 → 79
**Glufosinate D_3**	20	0.2	0.4	*m/z* 183 → 63
**MPPA D_3**	20	0.2	0.4	*m/z* 154 → 63
***N*-Acetyl-Glufosinate D_3**	20	0.2	0.4	*m/z* 225 → 63
**Cyanuric acid $^{13}C_3$**	20	0.2	0.4	*m/z* 131 → 43
**Chlorate $^{18}O_3$**	20	0.2	0.4	*m/z* 89 → 71
**Perchlorate $^{18}O_4$**	20	0.2	0.4	*m/z* 107 → 89
**Phosphonate $^{18}O_3$**	20	0.2	0.4	*m/z* 87 → 85
**Trifluoroacetic acid $^{13}C_2$**	10	0.1	0.2	*m/z* 115 → 70

Table S3: *Details on CE-MS/MS setup and measurement conditions employed in the final method of this study [2].*

**Sample preparation**					
Extraction	QuPPe-PO [1] or QuPPe-AO [3]				
Dilution of sample extract	5-fold in MeOH$_{FA}$*/H_2O 7/3**				
**CESI instrument settings**					
CESI instrument	Sciex CESI 8000 Plus ESI-MS-System				
Separation Capillary	Beckman Coulter OptmiMS Silica surface cartridge 30 µm ID x 91 cm				
Background electrolyte (BGE)	15/20/65 acetic acid/MeOH/water (*v*/*v*/*v*)				
Conductive liquid	10/90 acetic acid/ water (*v*/*v*)				
Focusing buffer	5 mM NH_4-acetate (pH 6.7)***				
Separation mode	30 kV; reversed polarity; 5 psi pressure				
**CESI separation procedure**					
Step	**Description**	**Pressure**	**Time**	**Direction**	**Voltage/ Solution**
Rinse 1	Conditioning	100 psi	2 min	forward	0.1 M HCl
Rinse 2		100 psi	2 min	forward	0.1 M NaOH
Rinse 3		100 psi	2 min	forward	BGE
Rinse 4		100 psi	2 min	Reverse	Conductive liquid
Inject 1	Injection	5 psi	10 s	forward	Focusing buffer
Inject 2		10 psi	20 s	forward	Sample
Inject 3		5 psi	10 s	forward	Focusing buffer
Inject 4		5 psi	30 s	forward	BGE
Separation	Separation	5 psi	16 min	Reverse polarity	30 kV
Ramp-down		5 psi	5 min	Reverse polarity	1 kV
**MS instrument settings**					
MS instrument	AB Sciex QTrap 5500				
Ion source	NanoSpray III Source				
Curtain gas (nitrogen)	5 psi				
Ion Spray Voltage	~ -2000 V				
Gas Flow	Off				
Temperature of Gas 2	50°C				

* MeOH$_{FA}$: Methanol containing 1% formic acid

** for the analysis of glufosinate, background electrolyte (BGE) was used as solvent for dilution

*** for the analysis of AMPA and glufosinate, no focusing buffer was used

Table S4: *CE-MS/MS mass transitions of the analytes, see also [1].*

**Compound**	**Mass Transition 1 (target/quantifier)**	**Mass Transition 2 (qualifier 1)**	**Mass Transition 3 (qualifier 2)**
Glyphosate	*m/z* 168 → 63	*m/z* 168 → 150	*m/z* 168 → 124
AMPA	*m/z* 110 → 63	*m/z* 110 → 79	*m/z* 110 → 81
NAGly	*m/z* 210 → 63	*m/z* 210 → 150	*m/z* 210 → 124
Fosetyl	*m/z* 109 → 81	*m/z* 109 → 63	*m/z* 109 → 79
Ethephon	*m/z* 143 → 107	*m/z* 145 → 107	*m/z* 143 → 79
HEPA	*m/z* 125 → 63	*m/z* 125 → 95	*m/z* 125 → 107
Glufosinate	*m/z* 180 → 63	*m/z* 180 → 95	*m/z* 180 → 85
MPPA	*m/z* 151 → 63	*m/z* 151 → 133	*m/z* 151 → 107
NAGlu	*m/z* 222 → 63	*m/z* 222 → 136	*m/z* 222 → 59
Chlorate	*m/z* 83 → 67	*m/z* 85 → 69	*m/z* 85 → 67
Perchlorate	*m/z* 99 → 83	*m/z* 101 → 85	*m/z* 99 → 67
Bromide**	*m/z* 81 → 81	*m/z* 79 → 79	
Phosphonate	*m/z* 81 → 79	*m/z* 81 → 63	
Trifluoroacetic acid	*m/z* 113 → 69	*m/z* 113 → 113*	

*no other useful mass transition available

**high collision energy was used to initiate complete fragmentation to any other (non-elemental) ions with the same *m/z* value [1]

Table S5: *Approximate injection volumes resulting in CE-MS/MS when applying the given pressure and time settings. Calculated with [4]*

**Pressure*time in psi*s**	**Injection volume in nL according to [4]**
50 psi*s = 5 psi * 10 s	~10 nL
150 psi*s = 10 psi * 15 s	~29 nL
200 psi*s = 10 psi * 20 s	~38 nL
400 psi*s = 10 psi * 40 s	~76 nL

Table S6: *pH-dependent microspecies of AMPA and glufosinate (Glu) according to chemicalize.com [5].*

pH range	Predominant microspecies	Net charge of predominant microspecies
0 - 7	AMPA ($-NH_3^+$, $-PO_3H^-$)	neutral
7 – 9.9	AMPA ($-NH_3^+$, $-PO_3^{2-}$)	-1
>9.9	AMPA ($-NH_2$, $-PO_3H^-$)	-2
0 – 3.8	Glu ($-NH_3^+$, $-PO_2H-CH_3$, $-COOH$)	+1
3.8 – 9.5	Glu ($-NH_3^+$, $-PO_2^--CH_3$, $-COO^-$)	-1
>9.5	Glu ($-NH_2$, $-PO_2^--CH_3$, $-COO^-$)	-2

Table S7: *Average CE-MS/MS recovery rates (n=5) and relative standard deviations (RSDs) of highly polar anionic pesticides in strawberry, milk, liver and soybean at the respective lowest successfully validated level. Results shown were calculated based on 2-point bracketing matrix-based calibration (on cucumber extracts) with the use of isotope-labelled internal standards (IL-ISs). All extracts were 5-fold diluted with* $MeOH_{FA}/H_2O$ *7/3 (v/v), except for glufosinate, which was diluted with background electrolyte (BGE).*

		**Strawberry**		**Milk**		**Liver**			**Soybean**	
**Analyte (target/quantifier CE-MS/MS mass transition, see Table S4)**	Spiking level in mg/kg	Average recovery (%)	RSD (%)	Average recovery (%)	RSD (%)	Average recovery (%)	RSD (%)	Spiking level in mg/kg	Average recovery (%)	RSD (%)
Glyphosate	0.05	138[c]	14	110	5	99	11	0.1	96	25[b]
AMPA	0.05	93	27[b]	94	7	96	12	0.1	n.d.[d]	-
NAGly	0.05	97	2	119	5	101	11	0.1	112	6
Glufosinate[a]	0.06	92	11	112	17	90	5	0.06	n.d.[d]	-
MPPA	0.02	103	5	92	5	83	11	0.04	92	32[b]
NAGlu	0.02	100	11	115	14	112	24[b]	0.04	79[c]	14
Ethephon	0.01	96	3	90	14	113	8	0.02	109	19
HEPA	0.02	106	4	91	12	n.a.[e]	-	0.04	110	9
Fosetyl	0.01	99	9	100	11	111	10	0.02	109	21[b]

[a] Results for glufosinate obtained using customized settings entailing dilution in background electrolyte (BGE) and injection without bracketing with a focusing buffer.

[b] Required performance criteria as regards repeatability (RSD ≤ 20%) not fulfilled in these cases (due to strong signal suppression and poor signal intensity).

[c] Average recovery outside the range (80-120%) where correction for bias may be omitted

[d] No peak detected at the envisaged level

[e] n.a.= not analysed. A proper validation of HEPA in liver at this spiking level was not feasible because of natural occurrence of HEPA in liver [6].

Table S8: *Average CE-MS/MS recovery rates (n=5) and relative standard deviations (RSDs) in lemon and milk at the respective lowest successfully validated level. Results shown were calculated based on 2-point bracketing matrix-based calibration (on cucumber extracts) with the use of IL-IS (except bromide). All extracts were 5-fold diluted with* $MeOH_{FA}/H_2O$ *7/3 (v/v).*

		Lemon			Milk	
**Analyte (target/quantifier CE-MS/MS mass transition, see Table S4)**	Spiking level in mg/kg	Average recovery (%)	RSD (%)	Spiking level in mg/kg	Average recovery (%)	RSD (%)
Bromide	5	118	8	5	115	7
Chlorate	0.03	118	8	0.03	107	7
Perchlorate	0.01	136[b]	12	0.02	113	37[a]
Phosphonate	0.05	107	5	0.05	97	7
TFA	0.05	n.a.[c]	-	0.025	n.a.[c]	-

[a] Required performance criteria as regards repeatability (RSD ≤ 20%) not fulfilled in these cases (due to strong signal suppression and poor signal intensity).

[b] Average recovery outside the range (80-120%) where correction for bias may be omitted

[c] Not evaluated due to residues in the used cucumber extracts.

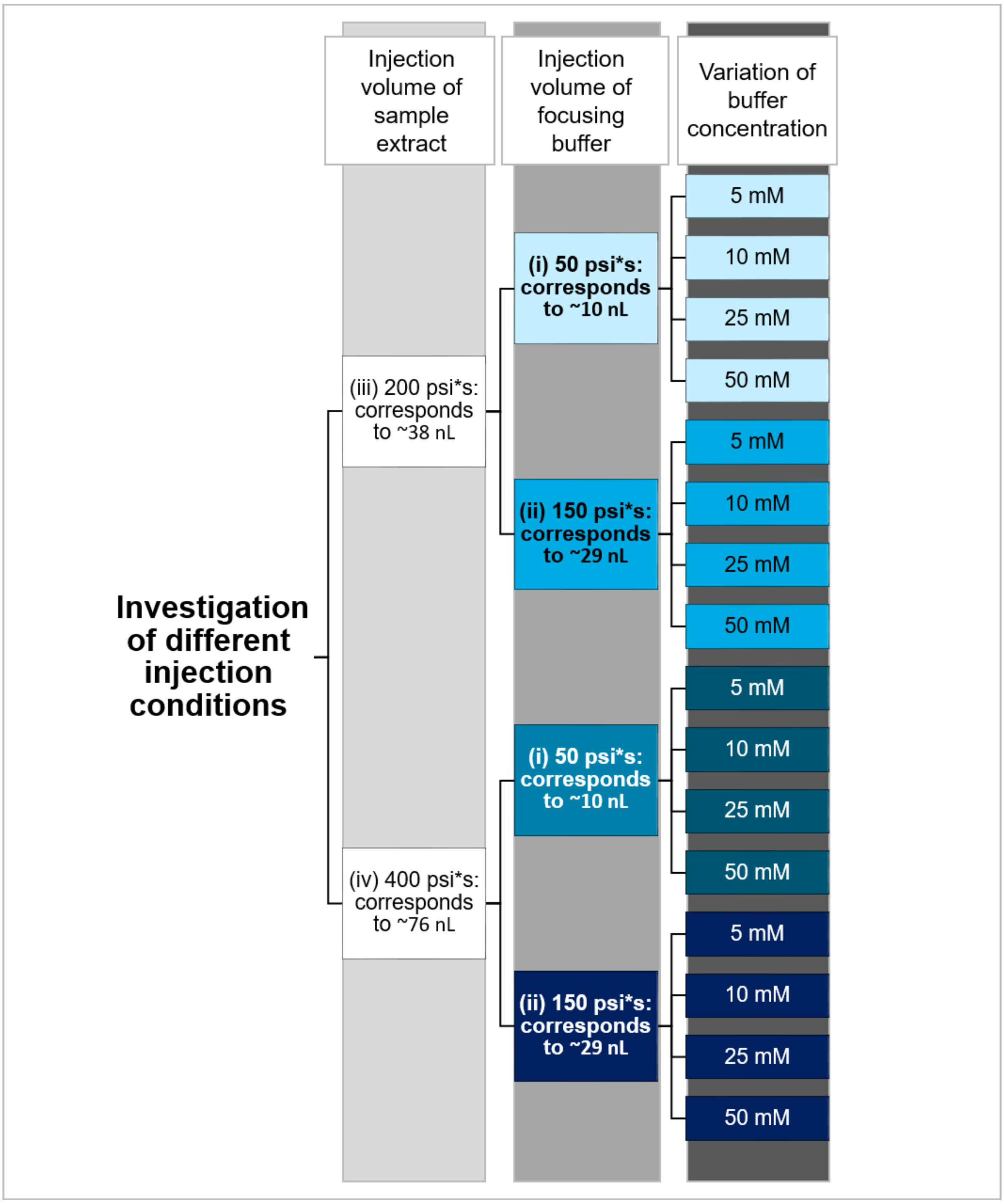

Fig. S1: *Scheme of the conducted experiments for the investigation of different injection conditions*

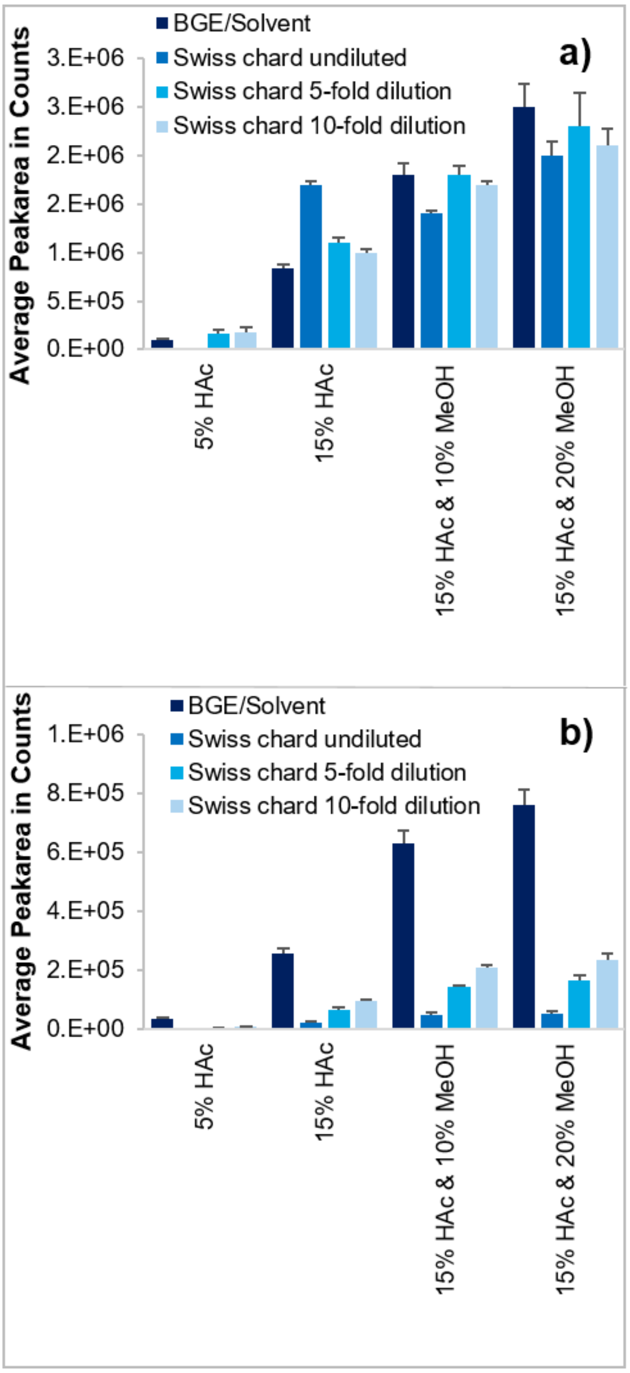

Fig. S2: *Comparison of average CE-MS/MS peak areas of glyphosate (**a**) and its main metabolite AMPA (**b**) at different compositions of background electrolyte (BGE). The two compounds were injected at 0.2 µg/mL in pure background electrolyte (BGE) (n=10), in undiluted QuPPe extract of Swiss chard (n=5) as well as in 5-fold (n=10) and 10-fold diluted (n=10) Swiss chard extract (dilution with the respective BGE). Of both compounds no signal was detected in undiluted extracts when using 5% HAc in water as BGE*

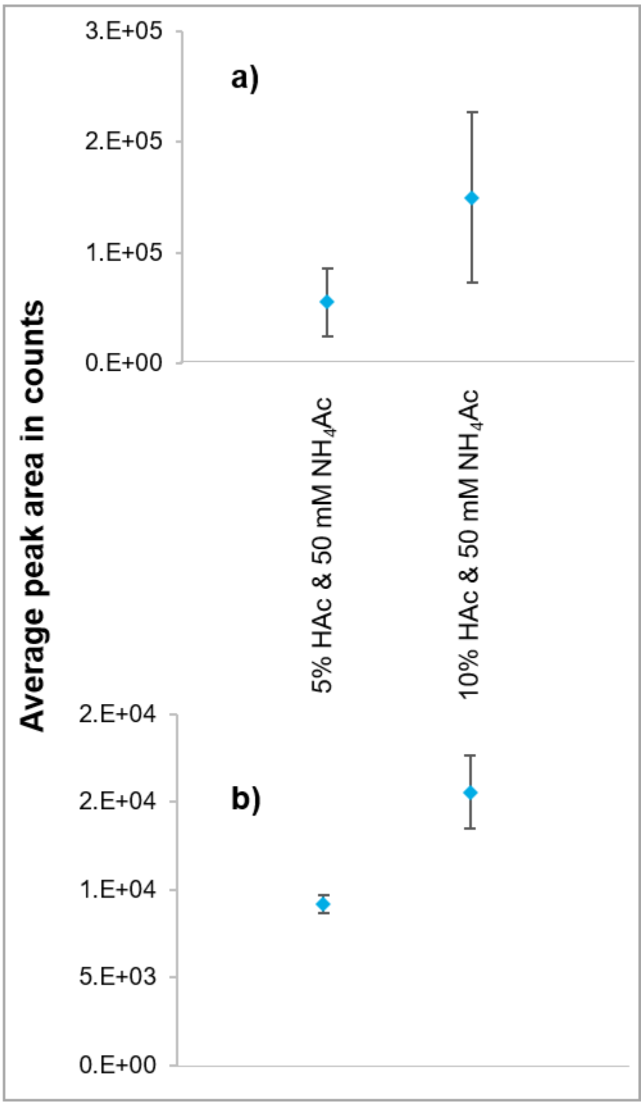

Fig. S3: *Average CE-MS/MS peak areas (n=10) of glyphosate (**a**) and its main metabolite AMPA (**b**) in a 5-fold diluted QuPPe extract of Swiss Chard (dilution with the respective background electrolyte) when using 50 mM NH_4 acetate (NH_4Ac) and a different share of HAc (5% and 10%) in the background electrolyte (BGE) [2]*

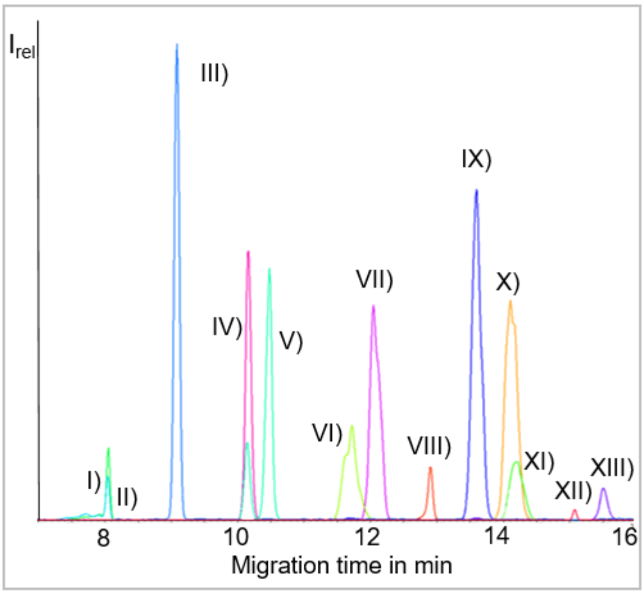

Fig. S4: *CE-MS/MS electropherogram of a standard mixture at 0.2 µg/mL in cucumber extract following 5-fold dilution with* $MeOH_{FA}$ *(MeOH containing 1% formic acid)/*H_2O *7/3 (v/v) [2]: I) perchlorate; II) chlorate; III) trifluoroacetic acid; IV) fosetyl; V) phosphonate; VI) N-acetyl-glyphosate; VII) ethephon; VIII) HEPA; IX) glyphosate; X) MPPA; XI) N-acetyl-glufosinate; XII) AMPA; XIII) glufosinate; bromide and phosphate not shown here*

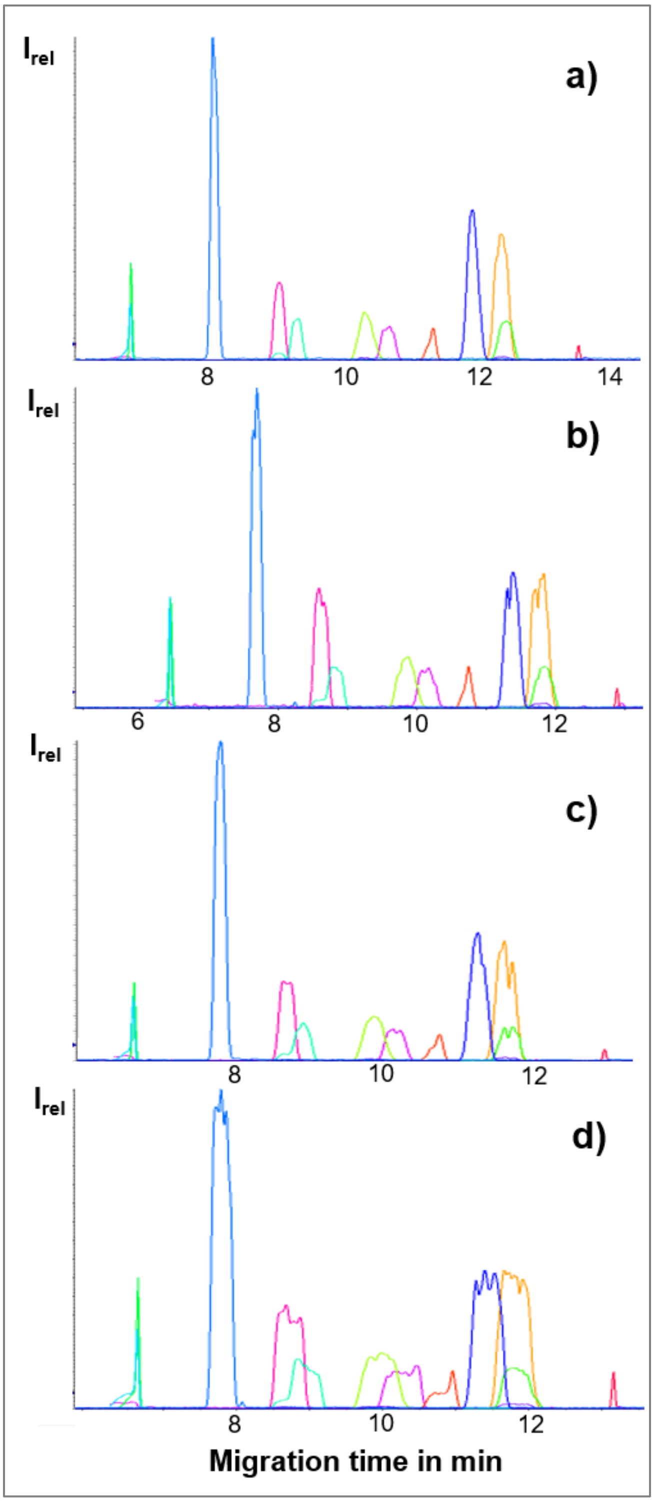

Fig. S5: *Exemplary CE-MS/MS electropherograms using different injection conditions:* ***a****) (iii) 200 psi*s injection of sample extract and (i) 50 psi*s injection of a 5 mM* NH_4*-acetate solution (final conditions);* ***b****) (iii) 200 psi*s injection of sample extract and (i) 50 psi*s injection of a 10 mM* NH_4*-acetate solution;* ***c****) (iii) 200 psi*s injection of sample extract and (ii) 150 psi*s injection of a 5 mM* NH_4*-acetate solution;* ***d****) (iv) 400 psi*s injection of sample extract and (i) 50 psi*s injection of a 5 mM* NH_4*-acetate solution. For colour assignment of peaks see* ***Fig. S4****, for an overview of the tested injection conditions, see* ***Fig. S1***

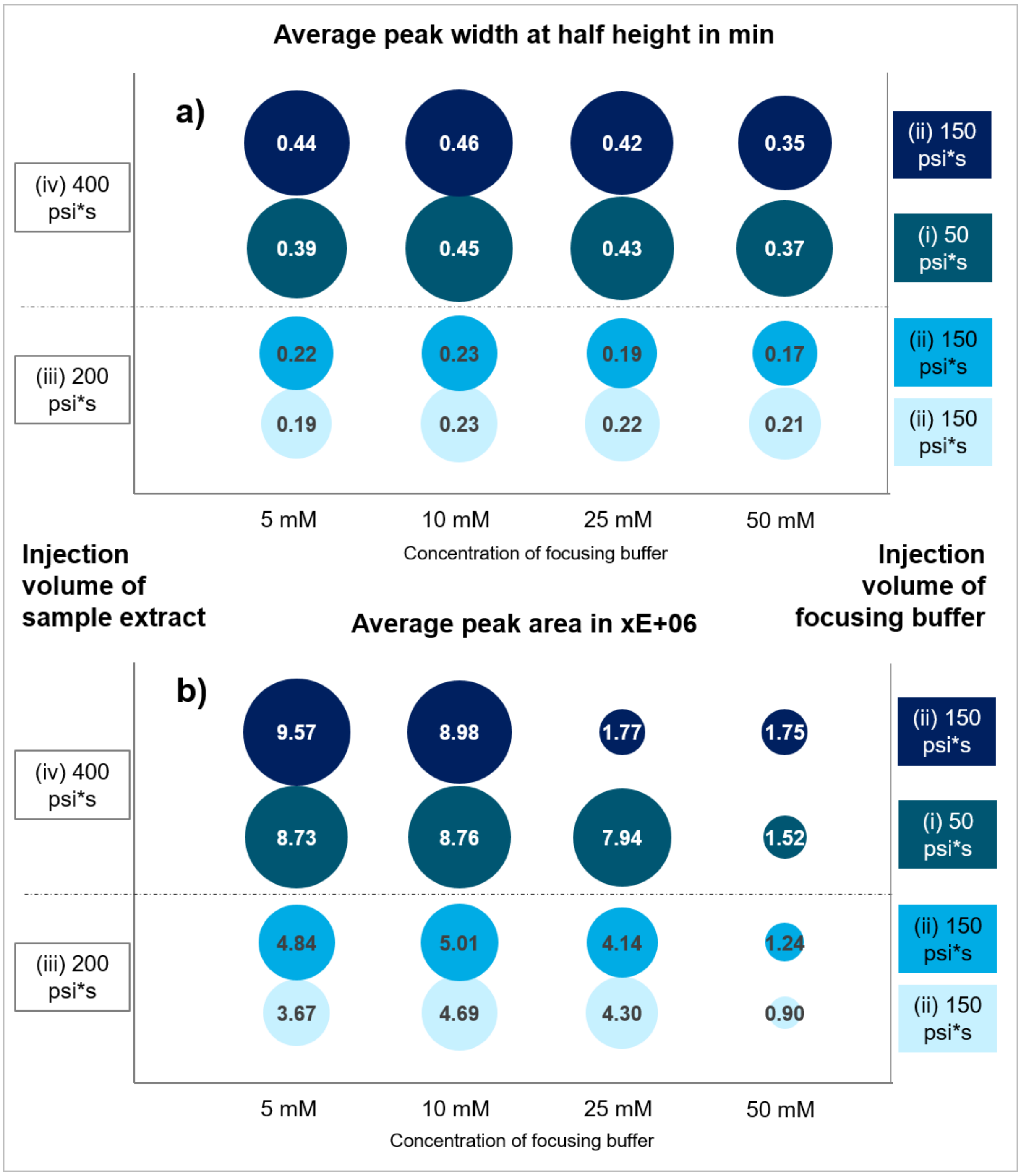

Fig. S6: *Average CE-MS/MS peak width at half height* ***a)*** *and average peak area* ***b)*** *displayed as bubble size according to the injected volume of sample (left axis, (iii) or (iv)), injected buffer volume (right axis, (i) or (ii)) and concentration of focusing buffer (x-axis)*

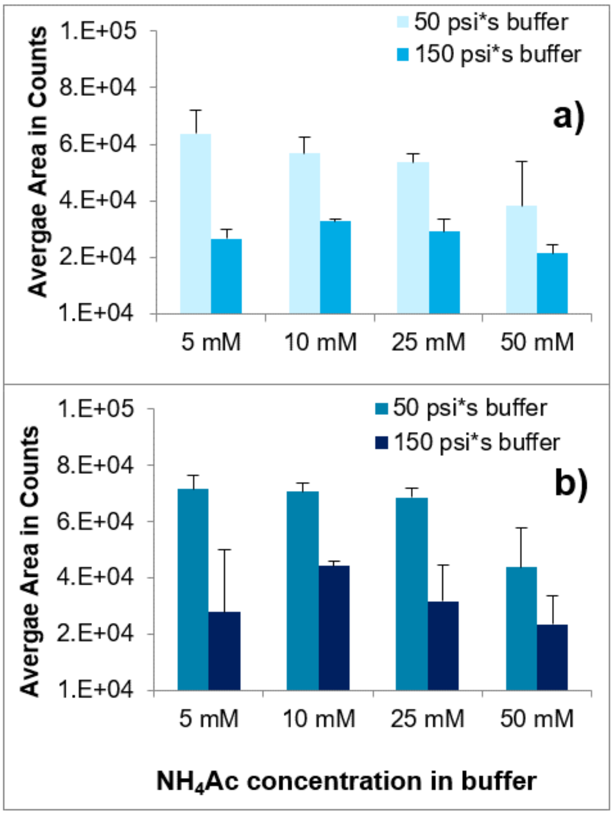

Fig. S7: *Comparison of average CE-MS/MS peak areas of AMPA (n=3) using 5 mM, 10 mM, 25 mM or 50 mM NH_4-acetate (NH_4-Ac) in water as buffer solution with (i) 50 psi*s (5 psi * 10 s) injection or (ii) 150 psi*s (10 psi * 15 s) injection of focussing buffer, each before and after sample extract injection. On the top, average areas with* ***a)*** *(iii) 200 psi*s (10 psi * 20 s) injection of sample extract between two injections of focusing buffer. On the bottom, average areas with* ***b)*** *(iv) 400 psi*s (10 psi * 40 s) injection of sample extract between two injections of focusing buffer*

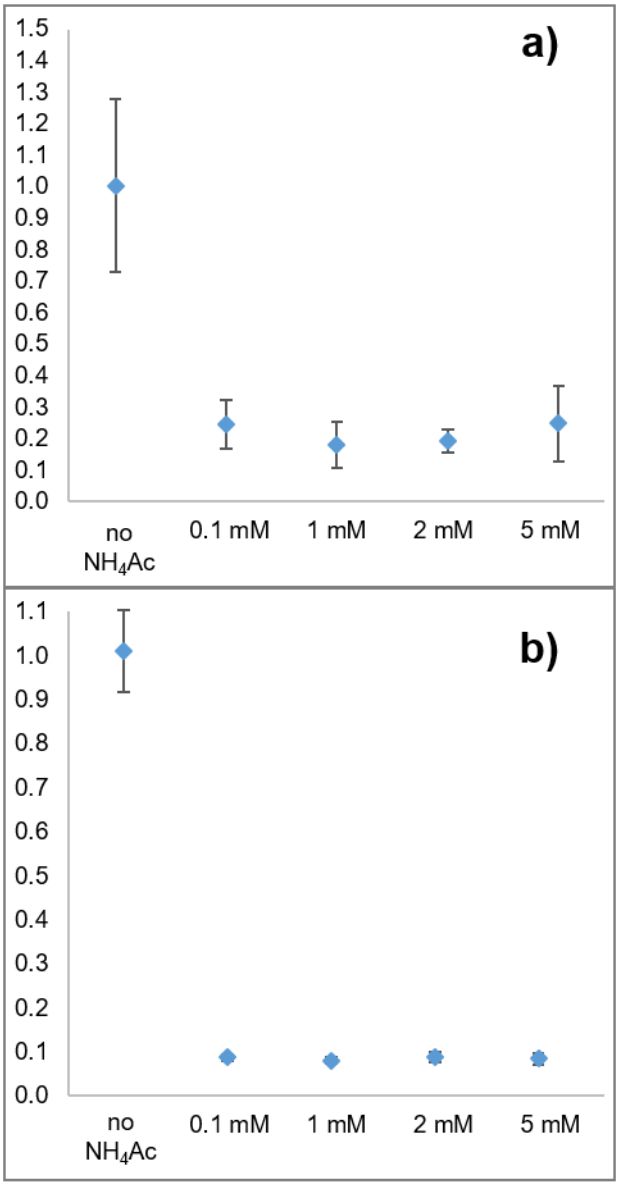

Fig. S8: *Signal loss when using NH_4-acetate as focusing buffer (0.1, 1, 2 and 5 mM) in the case of AMPA (**a**) and glufosinate (**b**). The average CE-MS/MS peak areas (n=3 each) when no buffer was used were normalized at a value of 1.0. The focusing buffer solution was injected with (i) 50 psi*s both before and after the injection of the sample extract*

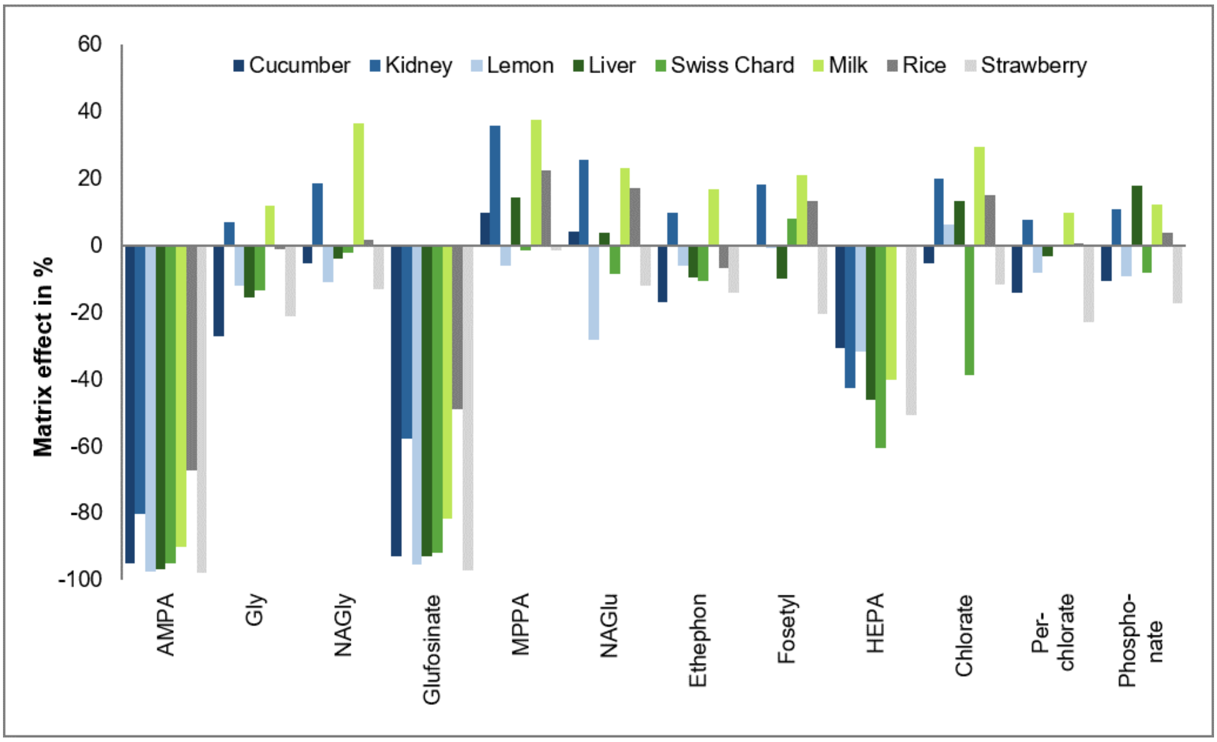

Fig. S9: *Matrix effects observed when injecting 5-fold diluted extracts of different matrices of plant and animal origin of all twelve studied compounds: glyphosate (Gly), glufosinate (Glu) and their metabolites (AMPA, N-acetyl-glyphosate (NAGly), MPPA and N-acetyl-glufosinate (NAGlu)), as well as ethephon, fosetyl, HEPA, chlorate, perchlorate, phosphonate* [2]*. '0 %' meaning no matrix effect, '-100 %' meaning total suppressions, matrix effect values >0 meaning signal enhancement*

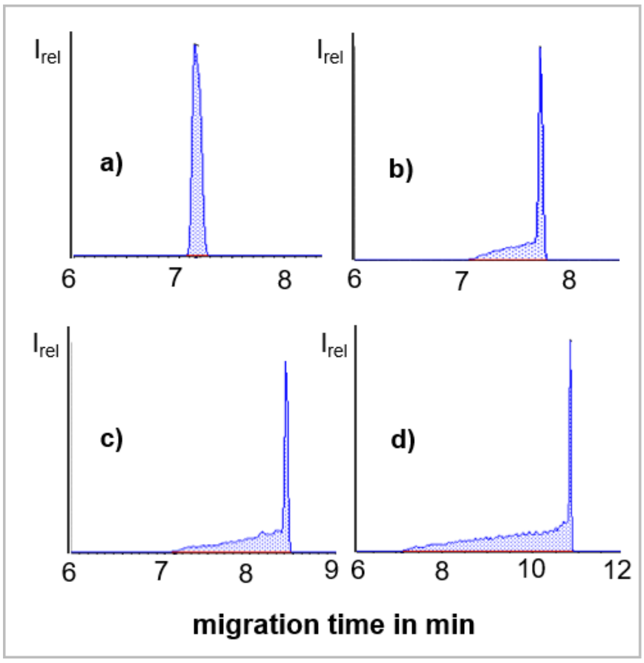

Fig. S10: *Shifts of migration time and peak shape (fronting) of perchlorate (at 0.2 µg/mL) in Swiss chard extracts when injected following dilutions at different rates: 20-fold (**a**), 10-fold (**b**), 5-fold (**c**) and undiluted (**d**)*

References

[1] Anastassiades M, Schäfer A-K, Kolberg DI, Eichhorn E, Dias H, Benkenstein A, Zechmann S, Mack D, Wildgrube C, Barth A, Sigalov I, Goerlich S, Cerchia G. Quick method for the analysis of highly polar pesticides in food involving extraction with acidified methanol and LC- or IC-MS/MS measurement – I. Food of plant origin (QuPPe-PO-Method) Version 12.3, 31.12.2024. https://www.eurl-pesticides.eu/userfiles/file/EurlSRM/EurlSrm_meth_QuPPe_PO_V12_3.pdf. Accessed Mar 2025.

[2] Wachtler A-K, Wildgrube C, Mack D, Barth A, Anastassiades M, Scherbaum E, Vetter W. Analysis of highly polar pesticides in food of plant and animal origin with CESI-MS/MS; 13th EPRW 2020, PD-04. https://www.eurl-pesticides.eu/userfiles/file/EurlSRM/EPRW%202020%20-%20PD04.pdf. Accessed Mar 2025.

[3] Anastassiades M, Schäfer A-K, Kolberg DI, Eichhorn E, Benkenstein A, Zechmann S, Mack D, Barth A, Wildgrube C, Sigalov I, Goerlich S, Dörk D, Cerchia G. Quick method for the analysis of numerous highly polar pesticides in food involving extraction with acidified methanol and LC-MS/MS measurement II. Food of animal origin (QuPPe-AO-Method); Version 3.3; 14.05.2019. https://www.eurl-pesticides.eu/userfiles/file/EurlSRM/EurlSrm_meth_QuPPe_AO_V3_3.pdf. Accessed Mar 2025.

[4] Free online calculator tool for fluid deliveries in CE provided by AB SCIEX: https://sciex.com/br/products/ce-expert-lite. Accessed Mar 2025.

[5] Chemicalize.com: https://chemicalize.com/welcome. Accessed Mar 2025.

[6] EURL-SRM: Residue findings of QuPPe-compounds in samples of plant origin from the German market in 2022. Version 1, 01.03.2023. https://www.eurl-pesticides.eu/userfiles/file/EurlSRM/EurlSrm_residue_findings_QuPPe-Compounds2022.pdf. Accessed Mar 2025.
